# Mapping model units to visual neurons reveals population code for social behaviour

**DOI:** 10.1038/s41586-024-07451-8

**Published:** 2024-05-22

**Authors:** Benjamin R. Cowley, Adam J. Calhoun, Nivedita Rangarajan, Elise Ireland, Maxwell H. Turner, Jonathan W. Pillow, Mala Murthy

**Affiliations:** 1 Princeton Neuroscience Institute, Princeton University, Princeton, NJ USA; 2 Cold Spring Harbor Laboratory, Cold Spring Harbor, NY USA; 3 Department of Neurobiology, Stanford University, Stanford, CA USA

**Keywords:** Computational neuroscience, Visual system, Social behaviour

## Abstract

The rich variety of behaviours observed in animals arises through the interplay between sensory processing and motor control. To understand these sensorimotor transformations, it is useful to build models that predict not only neural responses to sensory input^1–5^ but also how each neuron causally contributes to behaviour^6,7^. Here we demonstrate a novel modelling approach to identify a one-to-one mapping between internal units in a deep neural network and real neurons by predicting the behavioural changes that arise from systematic perturbations of more than a dozen neuronal cell types. A key ingredient that we introduce is ‘knockout training’, which involves perturbing the network during training to match the perturbations of the real neurons during behavioural experiments. We apply this approach to model the sensorimotor transformations of *Drosophila melanogaster* males during a complex, visually guided social behaviour^8–11^. The visual projection neurons at the interface between the optic lobe and central brain form a set of discrete channels^12^, and prior work indicates that each channel encodes a specific visual feature to drive a particular behaviour^13,14^. Our model reaches a different conclusion: combinations of visual projection neurons, including those involved in non-social behaviours, drive male interactions with the female, forming a rich population code for behaviour. Overall, our framework consolidates behavioural effects elicited from various neural perturbations into a single, unified model, providing a map from stimulus to neuronal cell type to behaviour, and enabling future incorporation of wiring diagrams of the brain^15^ into the model.

## Abstract (web summary)

A deep neural network with ‘knockout training’ is used to model sensorimotor transformations and neural perturbations of male *Drosophila melanogaster* during visually guided social behaviour and provides predictions and insights into relationships between stimuli, neurons and behaviour.

## Main

To understand how the brain transforms sensory information into behavioural action, an emerging and popular approach is to first train a deep neural network (DNN) model on a behavioural task performed by an animal (for example, recognizing an object in an image) and then compare the neural activity of the animal to the internal activations of the DNN^1–3,5,16,17^. A shortcoming of this approach is that the DNN does not predict how an individual neuron causally contributes to behaviour, making it difficult to interpret the role of the neuron in the sensorimotor transformation. Here we overcome this drawback by perturbing the internal units of a DNN model while predicting the behaviour of animals whose neurons have also been perturbed, a method that we call knockout training. This approach places a strong constraint on the model: each model unit must contribute to behaviour in a way that matches the causal contribution of the corresponding real neuron to behaviour. An added benefit is that the model infers neural activity from (perturbed) behaviour alone. This is especially useful when studying complex, natural behaviours, for which it can be challenging (or impossible in some systems) to obtain simultaneous recordings of neural activity. Here we use this approach to investigate the sensorimotor transformations of *Drosophila* males during natural social behaviours, including pursuit of and singing to a female^9^.

## A deep network model of vision to behaviour

**Fig. 1:**
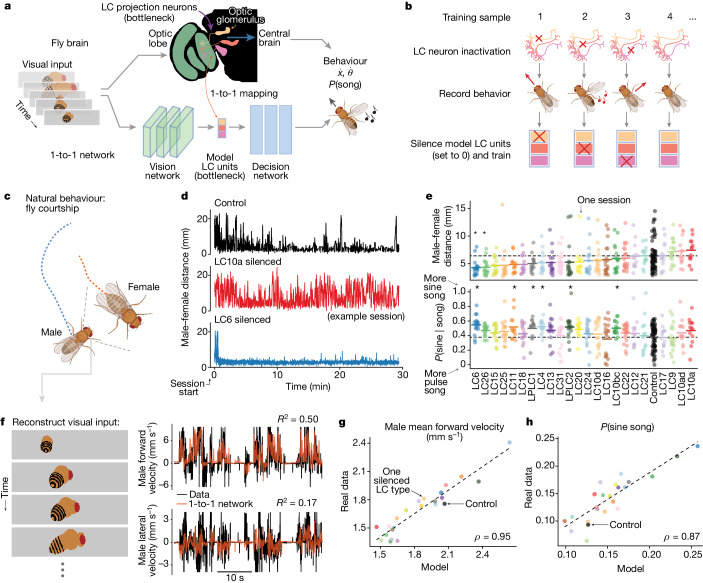
Identifying a one-to-one mapping between real neurons and internal units of a DNN with knockout training. **a**, We model the transformation from vision to behaviour in male flies with a DNN that comprises a bottleneck of model units to match the bottleneck of optic glomeruli in the visual system of the fly. We seek a one-to-one mapping in which one model unit corresponds to one optic glomerulus (innervated by a single LC neuron type) both in activity and in contribution to behaviour (for example, movement and song produced by wing vibration). **b**, We designed knockout training to fit this 1-to-1 network. After silencing an LC neuron type and recording the resulting behaviour, during training we ‘knocked out’ the model LC unit (that is, we set its activity value to 0 (red crosses)) corresponding to the silenced LC type. **c**, We (bilaterally) genetically inactivated males for each of 23 LC neuron types and then recorded the interactions of each male with a female during natural courtship. **d**, Courtship behaviour noticeably changed between control and LC-silenced male flies. Example sessions are shown. **e**, Changes in the average male-to-female distance following silencing of each LC type in males (top) and changes in the proportion of song that was sine versus pulse (bottom). Each dot denotes one courtship session. Short lines denote means; horizontal dashed line denotes mean of control sessions. Asterisks denote significant deviation from control. *P* < 0.05, permutation test, false discovery rate-corrected for multiple comparisons; *n* > 12. **f**, The 1-to-1 network takes as input an image sequence of the 10 most recent time frames (approximately 300 ms) of the visual experience of the male. Each image is a reconstruction of what the male fly observed based on male and female joint positions of that time frame (for example, **c**). The 1-to-1 network reliably predicts forward velocity (right, top), lateral velocity (right, bottom) and other behavioural variables (Extended Data Fig. 3) of the male fly. *R*^2^ values are from held-out frames across control sessions. **g**,**h**, The 1-to-1 network also reliably predicts overall mean changes in behaviour across males with different silenced LC neuron types, such as forward velocity (**g**) and sine song (**h**). Correlation *ρ* values were significant (*P* < 0.002, permutation test; *n* = 23). Source data

The *Drosophila* visual system contains a bottleneck between the optic lobes and the central brain in the form of visual projection neurons, which comprise approximately 200 different cell types^18,19^. The primary cell types of this bottleneck (Fig. 1a) are the 57 lobula columnar (LC) and lobula plate (LPLC) neuron types identified so far (we use ‘LC types’ to refer both to LC and LPLC neuron types), making up about 3.5% of all neurons in the brain. The LC neuron types receive input from the lobula and lobula plate in the optic lobe and send axons to optic glomeruli in the central brain^12,20^. Neurons of a single LC type innervate only one optic glomerulus in the posterior lateral protocerebrum, posterior ventrolateral protocerebrum or anterior optic tubercle neuropils, and prior studies have uncovered mappings between specific LC types, visual features and specific behaviours^11,21–28^. For example, LPLC2 neurons respond to a looming object and synapse onto the giant fibre neuron to drive an escape take-off^25^. LC11 neurons respond to small, moving spots and contribute to freezing behaviour^27,28^. For courtship, the LC10a neurons (and LC9 neurons, to a lesser extent) of a male participate in tracking the position of the female and driving turns towards the female^11,22,23^, but it is not yet known whether other LC types contribute to male social behaviours. As recordings from LC neurons reveal that even simple stimuli can drive responses in multiple LC types^29–31^, we explored whether the representation of the female during courtship might be distributed across the LC population, and similarly whether multiple LC types might be required to drive behaviour.

We designed a novel DNN modelling approach for identifying the functional roles of LC neuron types using behavioural data from genetically altered flies. The DNN model has three components: (1) a front-end convolutional vision network that reflects processing in the optic lobe; (2) a bottleneck layer of LC units in which each model LC unit represents the summed activity of neurons of the same LC type (that is, the overall activity level of an optic glomerulus); and (3) a decision network with dense connections that maps LC responses to behaviour, reflecting downstream processing in the central brain and ventral nerve cord (Fig. 1a). We imposed the bottleneck layer to have the same number of units as LC neuron types we manipulated, and our goal was to identify a one-to-one mapping between model LC units and LC neuron types. We did not incorporate biological realism into the vision and decision networks, opting instead for highly expressive mappings to ensure accurate prediction; we focused on explaining LC function. We collected training data to fit the model by blocking synaptic transmission^32^ in each of 23 different LC types in male flies^12,33^ and recorded the movements of the LC-silenced male and song production during natural courtship (Methods). We then devised a fitting procedure called knockout training, which involves training the model using the entire behavioural dataset of both perturbed and unperturbed sets of males. Critically, when training the model on data from a male with a particular LC type silenced (Fig. 1b), we set to 0 (that is, we knocked out) the activity of the corresponding model LC unit (correspondence was arbitrarily chosen at initialization; see Methods). The resulting model captures the behavioural repertoire of each genetically altered fly when the corresponding model LC unit is silenced, thereby aligning the model LC units to the real LC neurons. In simulations (Extended Data Fig. 2), knockout training correctly identified the activity and contribution to behaviour of each silenced neuron type (a one-to-one mapping) for neuron types that, when silenced, led to changes in behaviour. We refer to the resulting DNN model as the ‘1-to-1 network’.

**Extended Data Fig. 1:**
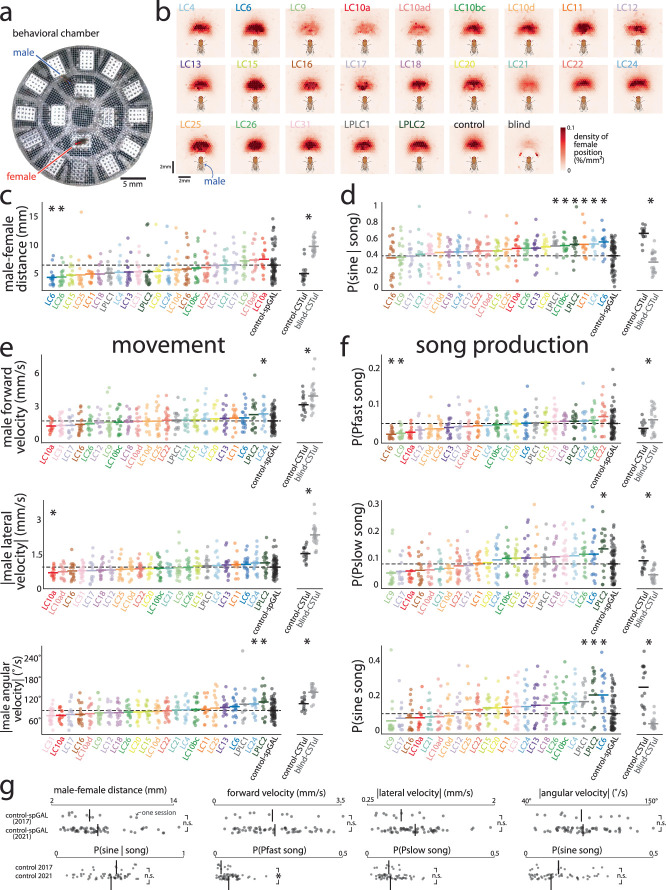
Different changes in behavior when silencing different LC neuron types of the male’s visual system. The main finding is that no single LC type showed a substantial change relative to control compared to the change observed between blind and control flies—suggesting no single LC type is the sole contributor to courtship behaviors. **a**. Image of circular behavioral chamber used to estimate the positions of a male (blue) and female (red) fruit fly during courtship. Joint positions for each frame were identified with the behavioral tracking software SLEAP^51^. Audio waveforms of song were detected with 16 microphones tiling the chamber (white boxes). **b**. Density of female position relative to the male’s egocentric view, conditioned on which LC type was silenced in the male as well as control-spGAL (‘control’) and blind-CSTul (‘blind’) males (multiple sessions per heatmap). Silencing any single LC neuron type did not extinguish courtship chasing (compare LC-silenced heatmaps to that of blind males); however, silencing some LC types did lead to a noticeable decrease in the amount of time females were positioned in front of the male versus control sessions (e.g., compare LC9, LC10a, LC10ad, and LC21 to control). **c**. Male-female distance averaged across the entire session for each silenced LC type (reproduced from Fig. 1e, top panel). Each dot is for one session; lines denote means and dashed line denotes the mean for control sessions. Statistically significant changes from control flies are indicated by an asterisk (*p* < 0.05, permutation test, corrected for the false discovery rate of multiple hypothesis testing by the Benjamini-Hochberg procedure, *n* > 12 for *n* sessions per LC type). We note that the spread across sessions (i.e., scatter of dots) per LC type is large; one likely reason for this spread is that the females were PIBL (pheromone insensitive and blind)—PIBL females tend to show larger individual differences in copulation time than wildtype females^9^. We also considered changes to behavior between control and blind male flies in CSTul flies (right, data from refs. ^9,52^ recorded in an 8-microphone arena, asterisks denote *p* < 0.05, permutation test, *n*≥15); the change in male-female distance between control and blind flies (an average of +4.80 mm) was substantially larger than the largest change between an LC type and control (for LC10a, an average of +1.03 mm; for LC6, −2.15 mm). Differences between our control-spGAL flies and control-CSTul flies are most likely due to the criteria for keeping a session (CSTul sessions were stopped and discarded if the male failed to begin courtship in the first 5 minutes; we did not have such restrictions for our control or LC-silenced sessions). Thus, only the relative changes between control-spGAL and LC-silenced sessions and the relative changes between control-CSTul and blind-CSTul should be compared. **d**. Proportion of sine song given song production. Same data as in Fig. 1e (bottom panel) except the LC types are ordered based on increasing proportions. Same format as in **a**. **e**. Mean changes in movement, including forward velocity (top panel), lateral velocity (middle panel), and angular velocity (bottom panel), averaged over the entire session. The absolute value was taken for lateral and angular velocity (i.e., speed), as we were interested in changes away from the male’s heading direction (e.g., a large turn to the right or left both indicated a large deviance). Same format as in **a**. **f**. Changes in the male’s song production, including the probability of sine, Pfast, and Pslow song. Same format as in **a**. Although we observed some significant changes in behavior (asterisks), overall we did not observe any LC types that, after silencing, resulted in changes to behavior on par with the changes observed between control and blind flies—opposite of what we were expecting if only one or two LC types were the dominant contributors to courtship. This suggests that multiple LC types need to be silenced together to obtain large deficits in behavior, consistent with our modeling results (Fig. 4). Previous studies have identified LC types LC10a and LC9 as contributing to courtship^11,22,23^, and our results are consistent: LC10a and LC9 show an increase in male-to-female distance (**c**, LC10a and LC9), as previously reported. A new implication for LC10a and LC9 is for song production: Both LC types tend towards a reduction in song production for all three song types (**f**, LC10a and LC9). The metrics we use here (e.g., taking the mean forward velocity across an entire session) are coarse summary statistics and do not represent all possible ways in which behavior may change due to silencing. In addition, variability across sessions per LC type was large, making it difficult to identify true changes. This motivated us to use the 1-to-1 network to model the LC-silenced and control behavior, as the 1-to-1 network can be used to directly identify the largest changes to the sensorimotor transformation due to LC silencing. In particular, we can use a metric—the coefficient of determination *R*^2^—that considers more possible changes than simply a change in mean offset. We use *R*^2^ when comparing changes to behavior for the 1-to-1 network (Fig. 4), but we cannot use *R*^2^ for the data here, as the visual inputs were not the same across silenced behavioral datasets. **g**. Our behavioral experiments comprised two sets of data collection that were 4 years apart, and we wondered if large deviations occurred for control-spGAL sessions between the two sets (both sets had the same genetic lines). We separated the control sessions into two groups (‘control 2017’ and ‘control 2021’, named for the year of collection) and found no significant difference between them across the movement and song statistics (n.s. denotes *p* > 0.05, permutation test) except for Pfast song (asterisk, *p* < 0.05, permutation test, *n* > 10). Thus, we felt confident in merging the two sets of data collection for further analysis. Source data

Before fitting the model with courtship data (Fig. 1c), we quantified the extent to which (bilaterally) silencing each LC neuron type changes behaviour of the male fly (Extended Data Fig. 1). Consistent with previous studies^11,23^, we found that silencing LC10a neurons resulted in failures to initiate chasing, as male-to-female distances remained large over time (Fig. 1d, middle,  1e, top); we found similar results with silencing LC9^22^. We also found strong effects on both chasing and singing when silencing other LC types. For example, silencing LC6 and LC26 neurons resulted in stronger and more persistent chasing, as male-to-female distances remained small over time (Fig. 1d, bottom,  1e, top). We observed a large number of LC types (LC4, LC6, LC11, LPLC1, LPLC2 and LC10bc) that, after silencing, significantly increased the amount of sine song relative to pulse song (Fig. 1e, bottom)—sine song typically occurs near the female^34^. Across behavioural measures, we found that the silencing of any single LC type did not match the behavioural deficits of blind flies (Extended Data Fig. 1). This suggests that many LC types would need to be silenced together to uncover large effects on courtship. We therefore modelled the perturbed behavioural data with the 1-to-1 network, enabling us to silence any possible combination of LC types in silico.

We performed knockout training to fit the parameters of the 1-to-1 network. The model inputs were videos of the visual input of the male fly during natural courtship (Methods and Fig. 1f, left); the model outputs comprised the male movements (forward, lateral and angular velocity) and song production, which included sine song and two forms of pulse song (Pfast and Pslow^35^). The 1-to-1 network reliably predicted these behavioural variables in held-out data (Fig. 1f, right and Extended Data Fig. 3). Notably, the 1-to-1 network also predicted differences in behaviour observed across silenced LC types (Fig. 1g,h and Extended Data Fig. 4). We confirmed that knockout training outperformed other possible training procedures, such as dropout training^36^ and training without knockout (that is, an unconstrained network) (Extended Data Figs. 3 and 4), and that results were largely consistent for different random initializations of the 1-to-1 network (Extended Data Fig. 5). Thus, the 1-to-1 network reliably estimated the behaviour of the male from visual input alone, even for male flies with a silenced LC type.

## Comparing real and model neural activity

**Fig. 2:**
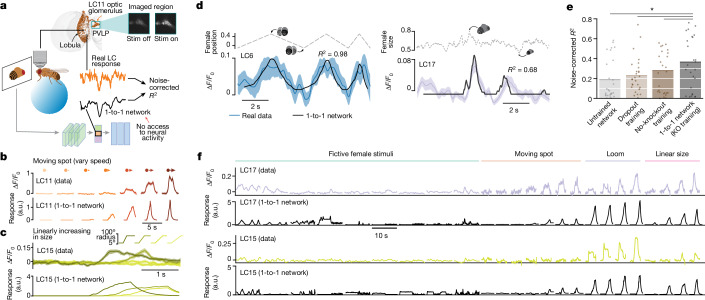
Model LC responses from the 1-to-1 network match real LC neural responses. **a**, We recorded LC responses using calcium imaging while a head-fixed male fly viewed dynamic stimulus (stim) sequences. We fed the same stimuli into the 1-to-1 network and tested whether the predicted responses (black trace) for a given model LC unit matched the real response of the corresponding LC neuron (orange trace, summed calcium dynamics within the region occupied by the glomerulus ‘imaged region’) by computing the noise-corrected *R*^2^ between the two (normalized) traces over time (Methods). The 1-to-1 network never had access to real LC responses during training, and only one pre-specified model LC unit was used to predict responses of each LC type. **b**, Real (top) and model (bottom) responses of LC11 to a moving spot with different speeds. a.u., arbitrary units. **c**, Real and model responses of LC15 to a spot with linearly increasing size. **d**, Real (colour traces) and model (black traces) LC responses to stimulus sequences of a fictive female changing in position and size (dashed traces). Shaded regions denote 90% bootstrapped confidence intervals of the mean; noise-corrected *R*^2^ values are indicated. **e**, Average noise-corrected *R*^2^ across all stimulus sequences and LC types for different networks (bars). Each dot denotes one LC type and stimulus pair. Dots with low *R*^2^ values primarily corresponded to weakly driving stimuli (Extended Data Fig. 8). The knockout network outperformed all other networks (*P < 0.05, paired, one-sided permutation test; *n* = 27). **f**, Real (colour traces, repeat-averaged responses) and model LC (black traces, unnormalized) responses across all presented artificial and natural stimuli. LC17 and LC15 are shown here; LC6, LC11 and LC12 responses are shown in Extended Data Fig. 7. Source data

**Extended Data Fig. 2:**
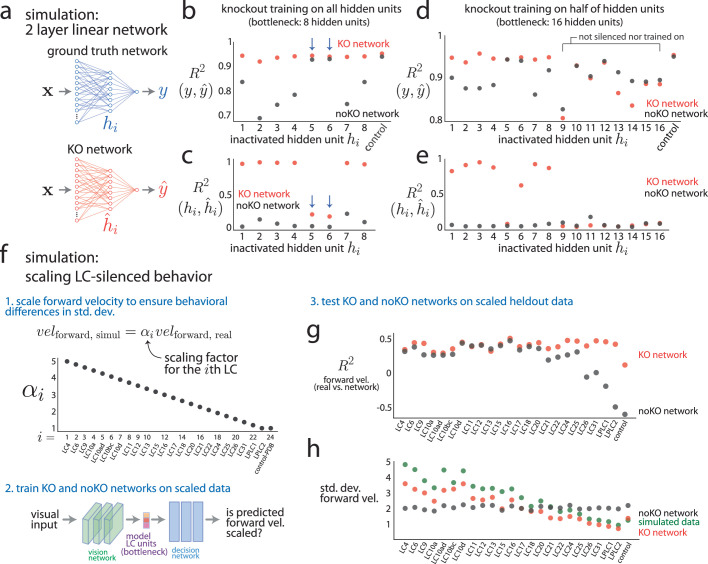
Testing the efficacy of knockout training with simulations. We tested the ability of knockout training to correctly identify the one-to-one mapping of silenced neuron types with two simulations. We compare a network trained with knockout (‘KO network’) to a network trained *without* knockout (‘noKO network’) for which no model units are inactivated (i.e., the noKO network has no knowledge that any silencing occurred). **a**. A simple simulation with 2 layer linear networks. The ground truth network (top) is a randomly-initialized, untrained 2 layer linear network with 48 input variables (${\bf{x}}\in {{\mathcal{R}}}^{48}$ where ${x}_{j} \sim {\mathcal{N}}(0,1)$), 8 hidden units (*h*_*i*_ for *i* = 1, …, 8), and 1 output unit (*y*). We use the same network architecture for the KO network (bottom). We seek a one-to-one mapping between the ground truth hidden units *h*_*i*_ and the model’s estimated hidden units ${\widehat{h}}_{i}$. We generated training data by silencing each hidden unit of the ground truth network (i.e., setting *h*_*i*_ = 0) and recording the resulting silenced output *y* as well as observing control data (for which no silencing occurred). For each case, we drew 1,000 input samples, which yielded 9, 000 training samples in total. We then trained the model either using knockout training (‘KO network’) or without it (‘noKO network’). We generated a test set in the same way as but independent of the training set; the test set also had 9, 000 test samples in total. **b**. We tested the KO network’s ability to correctly predict the silenced output *y* of the ground truth network. We collected the KO network’s predicted output $\widehat{y}$ to 1, 000 test samples for each silenced hidden unit of the ground truth network by knocking out the corresponding hidden unit in the KO network. We then computed the *R*^2^ (coefficient of determination) between *y* and $\widehat{y}$ for each silenced unit as well as control (red dots). We evaluated the noKO network with the same test set but did not knockout any hidden units during training or evaluation (black dots). We found that the KO network better predicted silenced output than that of the noKO network for most of the hidden units (red dots above black dots) but performance was roughly equal for control data (‘control’ red and black dots overlap). The KO and noKO networks had similar prediction performance for some of the silenced hidden units (*i* = 5 and 6, arrows); these units contributed little to the output of the ground truth network and, when silenced, led to outputs similar to those observed during control sessions. **c**. We then tested the KO network’s ability to correctly predict the hidden unit activity *h*_*i*_ for the *i*th hidden unit of the ground truth network (i.e., its “neural” responses). For the same test set as in **b**, we collected the KO network’s responses of its hidden units ${\widehat{h}}_{i}$ and computed the *R*^2^ (Pearson’s correlation squared) between *h*_*i*_ and ${\widehat{h}}_{i}$ (red dots). We performed the same evaluation for the noKO network (black dots) and found that the KO network substantially better predicted the activity of the ground truth’s hidden units versus the noKO network’s predictions (red dots above black dots). We observed some hidden units with low prediction performance both for the KO and noKO networks (*i* = 5 and 6, arrows). As expected, knockout training cannot identify mappings for these hidden units that contribute little to the ground truth network’s output (**b**, *i* = 5 and 6). Taking **b **and **c **together, we conclude that knockout training successfully identified the one-to-one mapping. **d**. We wondered to what extent does knockout training recover the one-to-one mapping when not all ground truth hidden units are silenced. This setting is more similar to our modeling of the fruit fly visual system, where we cannot silence all possible LC types. To test this, we gave the ground truth network and the model network each 16 hidden units (instead of 8 units) but only silenced the first 8 hidden units of the ground truth network (*i* = 1, 2, …, 8). We generated the training and test sets in the same manner as in **a**, ignoring the extra last 8 hidden units (*i* = 9, 10, …, 16), and trained the network with knockout training. The KO network correctly predicted output *y* for the first 8 silenced units (*i* = 1, 2, …, 8) but not for output resulting from silencing one of the last 8 units on which the KO network was not trained (*i* = 9, 10, …, 16, red and black dots overlap). The noKO network had worse prediction than that of the KO network for hidden units that contributed to the output (*i* = 1, 2, …, 8, black dots below red dots for most hidden units); inactivated hidden units with similar performance between KO and noKO networks (red dots and black dots overlap, *i* = 5) are due to the same reasons as that in **b **(arrows). **e**. Same as **c **except for 16 hidden units. As expected, the KO network recovers the activity for most of the first 8 hidden units (*i* = 1, 2, …, 8, red dots above black dots) but fails to recover the activity of the last 8 hidden units (*i* = 9, 10, …, 16, red and black dots close to *R*^2^ = 0). We note that the KO and noKO networks have similar poor performance ${R}^{2}({h}_{i},{\widehat{h}}_{i})$ for hidden unit 5 for the same reasons as the hidden units *i* = 5 and 6 in **c**. Taking **d **and **e **together, we conclude that knockout training still works to identify a one-to-one mapping (predicting both output/behavior and response) for hidden units that have been silenced—even if the remaining units in the bottleneck are never silenced. This motivates us to train the 1-to-1 network on behavioral data from silencing 23 LC types individually even though we do not have access to behavioral data from silencing the other remaining LC types in the bottleneck (57 LC types total in the bottleneck). **f**. Given that knockout training works in a simple simulation setting (**a**-**e**), we moved to testing knockout training for the 1-to-1 network used to model the fruit fly visual system (Fig. 1a). Although we could simulate data coming from a trained 1-to-1 network as ground truth, we were more interested in the case where there was a mismatch between the model and the real system—almost certainly the case using the 1-to-1 network to predict LC neuron types. Still, we sought some way to assess a ground truth change in behavior for the LC-silenced data and devised the following approach. For the *i*th LC type, we scale its forward velocity by *α*_*i*_, where *α*_*i*_ decreases from 5 to 1 as index *i* increases incrementally. We then train a KO network on this scaled data in the same manner as that of the 1-to-1 network; we also train a noKO network that has no knowledge if its training sample comes from LC-silenced or control males. We only train the networks to predict forward velocity (no other behavioral outputs). **g**. We computed prediction performance *R*^2^ (coefficient of determination) between predicted and actual forward velocity on held-out frames for each LC-silenced behavior. The KO network had better prediction than that of the noKO network both for the most scaled and least scaled LC types (red dots above black dots for leftmost and rightmost dots). **h**. This change in performance between KO and noKO networks for the most and least scaled LC types in **g **can be explained by how the KO and noKO networks each predict the standard deviation of forward velocity. As expected from our scaling of the real data (**f**), the standard deviation of the simulated data linearly falls as we consider the later LC types (green dots, compare with black dots in **f**). We find that the standard deviations predicted by the KO network also linearly decrease (red dots) while those predicted by the noKO network remain relatively flat (black dots). Because the noKO network has no information about which LC type was silenced, the noKO network must predict roughly the same standard deviation for all LC types, choosing an intermediate standard deviation (around 2 s.d.). This also helps to explain why the KO and noKO networks differed in prediction more for the rightmost LC types (**g**, ‘LC26’ to ‘control’) because the noKO network overestimated the standard deviation for these LC types (black dots above green dots for ‘LC26’ to ‘control’) leading to larger errors (and a negative coefficient of determination) versus underestimating the standard deviation which does not lead to as large drops in *R*^2^ (**g**, LC4 to LC10d). These simulations show that knockout training can reliably identify one-to-one mappings between model units and internal units given behavior resulting from silencing those internal units. Source data

One prediction from our simulations (Extended Data Fig. 2) is that the knockout training procedure, which leverages natural behavioural data only, should nonetheless learn the visual responses of real LC neurons. We recorded LC calcium dynamics in head-fixed, passively viewing male flies walking on an air-supported ball (Fig. 2a and Methods). We targeted 5 different LC neuron types (LC6, LC11, LC12, LC15 and LC17), chosen because silencing each one led to noticeable changes in courting behaviour (Fig. 1e and Extended Data Fig. 1). We first presented artificial stimuli (Fig. 2b,c and Methods) used to characterize LC responses in previous studies^29–31^. Despite the fact that the 1-to-1 network never had access to neural data, we found that its predicted responses largely matched their corresponding real LC responses for artificial stimuli (Fig. 2b,c, compare top and bottom, and Extended Data Fig. 7).

We then tested the predictions of the 1-to-1 network on more naturalistic stimulus sequences (that is, a fictive female varying her position, size and rotation; Supplementary Video 1). We found that the recorded LC neurons responded to many of these naturalistic stimulus sequences (Fig. 2d, colour traces, and Extended Data Fig. 8) and found reliable matches between real LC responses and their corresponding model LC responses (Fig. 2d, black traces versus colour traces, and Extended Data Fig. 8), yielding an average noise-corrected *R*^2^ of approximately 0.35. This was a significant improvement over other networks with the same architecture but trained with dropout or without knockout procedures (Fig. 2e); training on behaviour was important for prediction, as these networks outperformed an untrained network (Fig. 2e, untrained). The prediction performance of the 1-to-1 network was consistent with our expectations—exact matches were unlikely owing to differences in behavioural state during courtship (on which the 1-to-1 network was trained) and during imaging^11,31^.

**Extended Data Fig. 3:**
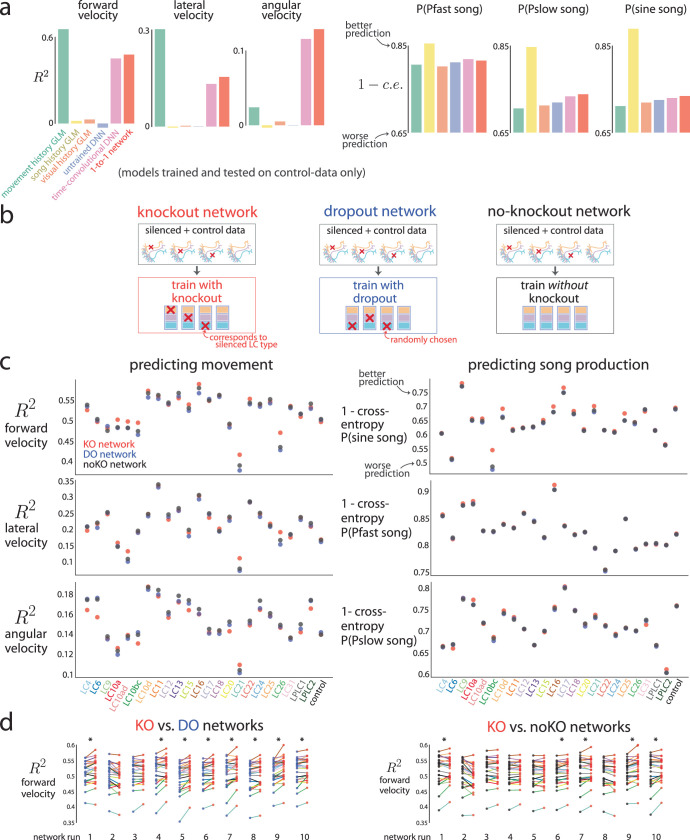
Predicting behavior frame-by-frame. Here we compare the extent to which the 1-to-1 network better predicts frame-by-frame behavior versus other network architectures and baseline models as well as other training procedures. **a**. We considered different network architectures for the 1-to-1 network and compared their prediction performance to baseline models. We trained each model on control sessions only and tested on held-out test frames of control sessions. For baseline models, we considered a generalized linear model (GLM) that took as input the last 300 ms of movement history, including forward, lateral, and angular velocity (‘movement history GLM’); past song history, including Pfast, Pslow, and sine song (‘song history GLM’); as well as the male’s past visual history represented by female size, position, and rotation (‘visual history GLM’). The movement-history-GLM had good prediction of forward and lateral velocity (two leftmost plots), as expected, but failed at predicting angular velocity and song production. Its good prediction (*R*^2^ > 0.6 for forward velocity) stems from the fact that an animal’s forward velocity at time step *t* is likely similar to its forward velocity at time step *t* − 1 based on the physics of movement. Likewise, the song history GLM best predicted song production (three rightmost plots), as songs often occur in bouts, but failed at predicting moment-to-moment movement (three leftmost plots). Also expected was the poor prediction performance of the visual-history-GLM, whose inputs of the fictive female’s parameters likely must pass through a strong nonlinear transformation to accurately recover behavior (all orange bars are low). Next, we considered the DNN architecture of the 1-to-1 network (Fig. 1a). We trained the 1-to-1 network on control data only (i.e., no knockout training was performed) for this analysis. The 1-to-1 network’s prediction performance was better than any GLM model for angular velocity and showed good performance for song production (red bars). The 1-to-1 network did not outperform the movement-history-GLM on forward and lateral velocity; providing past movement history to the 1-to-1 network is an intriguing direction not investigated in this work. We confirmed that an untrained network with the same architecture as the 1-to-1 network (‘untrained DNN’, only its last readout layer was trained) had little prediction ability. Finally, we trained a more complicated version of the 1-to-1 network which had 3-d convolutions in both space (2-d) and time (1-d) in the vision network (‘time-convolutional DNN’ with 3 × 3 × 3 convolutional kernels). This greatly increased the number of parameters but ultimately did not improve prediction performance versus the 1-to-1 network (pink versus red bars). We suspect that with more data, the time-convolutional DNN will outperform the current architecture of the 1-to-1 network, as motion processing occurs before the LC bottleneck^46^. **b**. As a test of the 1-to-1 network’s ability to uncover a one-to-one mapping between model LC units and real LC neurons, we tested the extent to which the 1-to-1 network accurately predicts behavior on held-out courtship frames for each silenced LC type. An important comparison is to measure the 1-to-1 network’s prediction performance relative to networks with the same architecture and training data but with different training procedures. Here, we illustrate three different training procedures. Knockout training (left, red) sets to 0 the model LC unit that corresponds to the LC neuron type silenced for that training frame (no model LC units’ values are set to 0 for frames from control sessions). We refer to the resulting trained network as the knockout (KO) network or, interchangeably, as the 1-to-1 network. Dropout training^36^ (middle, blue) sets to 0 a randomly-chosen model LC unit for each training frame, independent of the frame’s silenced LC type (no model LC units’ values are set to 0 for frames from control sessions). In this case, the number of ‘dropped out’ units equals that of the ‘knocked out’ units. We refer to the resulting trained network as the dropout (DO) network. Finally, we train a network *without* knocking out any of the model LC units and refer to it as the noKO network (right, black). The DO and noKO networks are appropriate controls (i.e., null hypotheses) for the KO network. The DO and noKO networks have no knowledge that any LC silencing has occurred; in other words, the DO and noKO networks assume all male flies, regardless of an LC type being silenced or not, have the same behavioral output to the same input stimulus. Thus, the DO and noKO networks cannot reliable detect changes in behavior for different silenced LC types unless the statistics of the visual input itself differs across silenced LC types. The latter may occur if, for example, silenced flies do not chase the female, the female will be visually smaller for most frames, leading DO and noKO networks to correctly predict a decrease in song production (as song is produced in close proximity to the female). **c**. We tested the KO, DO, and noKO network’s performance of predicting the male fly’s movement (left) and song production (right) for the next frame given the 10 past frames of visual input (a period of 300 ms) across many LC-silenced and control flies (459 sessions in total). All test frames were held out from any training or validation sets and sampled randomly in 3 s time periods across sessions (27,000 test frames per each LC type and control, see Methods). We computed the coefficient of determination *R*^2^ for behavioral outputs of movement (forward, lateral, and angular velocity) and 1 - binary cross-entropy (where a value close to 1 indicates good prediction) for behavioral outputs of song production (probabilities of Pfast, Pslow, and sine song). We found that overall, the KO network better predicts forward velocity than the DO and noKO networks (top left, red dots above black and blue dots) as well as the probability of sine song (top right). Changes in prediction performance between KO and DO/noKO networks across LC types were relatively small, suggesting changes in behavior were subtle, consistent with overall mean changes in behavior (Extended Data Fig. 1). In addition, *R*^2^ may change little for large second-order changes in behavior, such as variance (Extended Data Fig. 2g, leftmost dots). We confirmed in Fig. 1g–h and Extended Data Fig. 4 that the KO network accurately predicted mean changes in behavior better than DO and noKO networks. We note that *R*^2^ values for movement (left column, *R*^2^ ≈ 0.5 for forward velocity, *R*^2^ ≈ 0.15 for lateral and angular velocity) were not close to 1 because we predict rapid changes to movement variables frame-to-frame (with a frame rate of 30 Hz). Because the 1-to-1 network is deterministic (i.e., returning the same output for the same visual input), it fails to account for the fact that a male fly’s moment-to-moment decision is stochastic—in other words, the male responds differently to repeats of the same stimulus sequence. To take this stochasticity into account, one would need to present identical repeats of the same visual stimulus sequence and record the resulting behavior. This is not possible for our natural courtship experiments, where a male fly’s visual experience is determined by his behavior. However, this may be possible in future experiments using virtual reality, where the experimenter has greater control over a male fly’s visual input. **d**. Results in **c **were for a KO network with one random initialization. To see if this effect holds for different initializations, we trained 10 runs of the KO network, each with a different random initialization and random ordering of training samples. We found that for 8 of the 10 runs, KO networks outperformed DO networks (left); 5 of the 10 runs, KO networks outperformed noKO networks (right) in predicting forward velocity. For each run (i.e., ‘network run 1’), the same randomly initialized network and randomized order of training samples was used as a starting point for the KO, DO, and noKO network. Each connected pair of dots denotes one LC type with the color of the line connecting two dots denoting the LC type identity (same colors as in **c**). An asterisk denotes a significant difference in means (*p* < 0.05, paired, one-sided permutation test, *n* = 23). Network run 1 was chosen as the 1-to-1 network in **c **as well as Figs. 1–4 due to its high prediction for both behavior and neural responses. Source data

We further tested the predictions of the 1-to-1 network by assessing the extent to which the 1-to-1 network predicted response magnitudes across both natural and artificial stimuli and found reasonable matches (Fig. 2f and Extended Data Fig. 7). We also gave the 1-to-1 network partial access to neural data by using real LC responses to fit a linear mapping between all model LC units and one real LC neuron type. We found that held-out prediction improved to a noise-corrected *R*^2^ of approximately 0.65 (Extended Data Fig. 8), suggesting that better alignments between the model LC units and real LC types exist, at least for neural prediction. The 1-to-1 network was the most consistent in its neural predictions (across ten different random initializations) compared with other training procedures (Extended Data Fig. 6), suggesting that knockout training converges to a similar solution despite a different initialization. There are yet additional ways to test the model: by silencing or activating combinations of LC types predicted by the model to act in concert or by recording from LC types under conditions more similar to natural courtship. Nevertheless, we interpret our tests of the model to suggest that the 1-to-1 network has learned a reasonable mapping between visual stimulus and an individual LC type as well as the contribution of an individual LC type to behaviour. The sections that follow examine the 1-to-1 network that led to the best prediction of both behaviour and neural responses (of the ten different initializations; Extended Data Figs. 3 and 8).

## Visual feature encoding of the model LC units

**Fig. 3:**
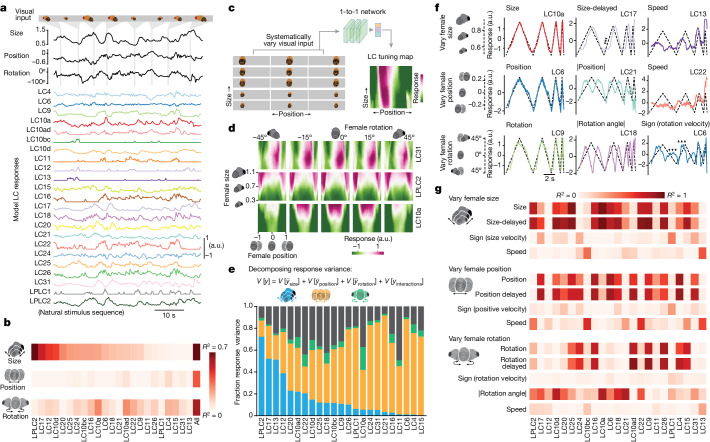
Visual features of female motion are distributed across the population of model LC units. **a**, Almost all model LC units responded to a fictive female changing in size, position and rotation. **b**, Cross-validated *R*^2^ between each primary visual parameter and model LC responses for natural stimulus sequences. Columns are sorted based on female size (top). The end column of each row (all) is the cross-validated *R*^2^ between a linear combination (identified via ridge regression) between all model LC units and a single visual parameter. **c**, We characterized the tuning preferences of each model LC unit by systematically varying the three visual parameters and computing a heat map of the model LC responses. Each input sequence was static (that is, all ten frames were repeats of the same image). **d**, Tuning heat maps for example model LC units (see Extended Data Fig. 9 for all LC types). **e**, We used variance decomposition (Methods) to decompose the response variance *V*[*y*] of each model LC unit into components solely due to either female size $V[\,{\bar{y}}_{{\rm{size}}}]$ (blue), position $V[\,{\bar{y}}_{{\rm{position}}}]$ (orange) or rotation $V[\,{\bar{y}}_{{\rm{rotation}}}]$ (green) as well as interactions between these visual parameters *V*[*y*_interactions_] (black). A large fraction of response variance for a given parameter indicates that a model LC unit more strongly changes its response *y* to variations in this parameter relative to those in other parameters. Because the 1-to-1 network is deterministic, all response variance can be attributed to variations of the parameters (that is, there is no repeat-to-repeat variability). **f**, Example model LC responses to dynamic stimulus sequences in which the fictive female solely varied either her size, position or rotation angle over time (dashed traces). Different model LC units appear either to directly encode a visual parameter (for example, LC10a encodes size) or encode features derived from the parameter, such as a delay (LC17, arrows) or speed at which female size changes (LC13). Responses for all model LC units are in Extended Data Fig. 10. **g**, *R*^2^ between model responses and visual parameter features for the stimulus sequences in **f**. Columns are in the same order as those in **b**. Source data

We next tested how the population of 23 model LC units encodes the movements of the female. We found that the majority of model LC units in the 1-to-1 network responded to changes in female position, size and rotation (Fig. 3a). Moreover, almost no model LC unit directly encoded any single visual parameter (Fig. 3b, low *R*^2^ values for any one LC type, but high *R*^2^ for a linear mapping of all LC types).

Males pursue females at a range of distances and positions, and we can use the 1-to-1 network to uncover how the LC population encodes these contexts by examining 3D ‘tuning maps’ (Fig. 3c, Extended Data Fig. 9 and Methods). Some model LC units, such as LC31, were driven by the position of the female (in front of the male), independent of female size and rotation (Fig. 3d, top), whereas other model LC units, such as LPLC2, were driven by large female sizes, consistent with its known response to looming stimuli^17,24,25,31^. Model LC10a was driven by female position (in front of the male), consistent with prior work^11,23^, but we found this was only true for conditions in which he is close and directly behind her (Fig. 3d, bottom). Model LC9 and LC22 were similarly driven by females in front of and facing away from the male, but at larger distances (Extended Data Fig. 9).

To quantify these interactions, we decomposed the response variance^37^ of each model LC unit into four components (Fig. 3e). Most model LC units encoded changes in female position (Fig. 3e, orange bars), roughly half encoded female size (Fig. 3e, blue bars), and female rotation was weakly encoded (Fig. 3e, green bars are small). However, almost all model LC units encoded some nonlinear interaction among the three visual parameters (Fig. 3e, black bars; on average around 25% of the response variance for each model LC unit).

We next considered non-naturalistic stimulus sequences, varying one visual parameter at a time (Fig. 3f, dashed lines, and Supplementary Video 2). For example, we varied the size of the female over time at different speeds, while keeping her position and rotation constant (Fig. 3f, top, dashed lines). For this stimulus, some model LC units perfectly encoded female size (Fig. 3f, top left, LC10a), some model LC units encoded a time-delayed version of size (Fig. 3f, top, middle, LC17), whereas other model LC units encoded the speed at which female size changed (Fig. 3f, top right, LC13). Similar relationships were present for other stimulus sequences and model LC units (Fig. 3f, bottom two rows); we note that the 1-to-1 network was predictive of real LC responses for similar types of stimulus sequences (Fig. 2d,e and Extended Data Fig. 8).

Compiling these results, we find that most model LC units encode some aspect of female size, position and rotation (Fig. 3g). Our results were consistent with previous studies, such as LC11 encoding the position of a small moving spot^27,28^ (Fig. 3g, LC11 has highest *R*^2^ for ‘position’ in ‘vary female position’ than in other stimulus sequences) and LPLC2 encoding loom^24^ (Fig. 3g, LPLC2 has highest *R*^2^ for ‘size’ in ‘vary female size’). Recently, LPLC2 has also been found to encode the speed of a moving spot^38^, consistent with the predictions of our model (Fig. 3g, LPLC2 has high *R*^2^ for ‘speed’ in ‘vary female position’). Model units LC4, LC6, LC15, LC16, LC17, LC18, LC21 and LC26 all encode female size (Fig. 3g, top), matching recent findings that these LC neurons respond to looming objects of various sizes^29–31^; our 1-to-1 network also uncovers that these LC types probably encode other visual features as well. Of note, results differed between varying a single female parameter versus combinations of parameters (compare with Fig. 3b,g); this highlights the importance of using more naturalistic stimuli to probe the visual system.

We conclude that the model LC units encode visual stimuli in a distributed way: each visual stimulus feature is encoded by multiple model LC units (Fig. 3g, rows each have multiple red squares), and each model LC unit encodes multiple visual stimulus features (Fig. 3g, columns each have multiple red squares). Consistent with this, the response-maximizing stimulus sequence for each model LC unit strongly drove responses of other model LC units, even when optimized for these other responses to be suppressed (a ‘one hot activation’; Extended Data Fig. 11).

## Linking model LC units to behaviour

**Fig. 4:**
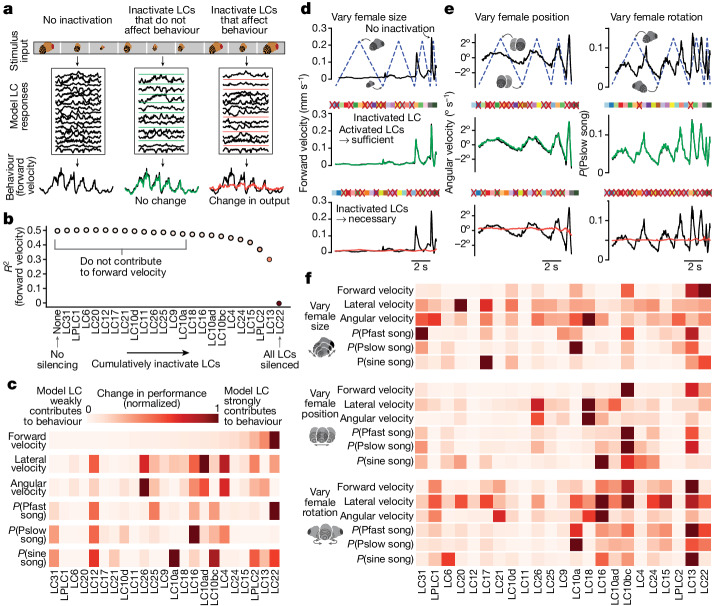
Combinations of model LC units are required for behaviour. **a**, We assess whether a group of model LC units are sufficient and necessary for behaviour if we inactivate all model LC units not in that group (middle, sufficient) or inactivate only that group of model LC units (right, necessary). **b**, We identify which model LC units contribute to forward velocity by cumulatively inactivating model LC units in a greedy manner (that is, inactivate the next model LC unit that, once inactivated, maintains the best prediction performance *R*^2^). The model LC units with the largest changes in performance (for example, LC13 and LC22) contribute the most. **c**, Results for cumulative inactivation for all six behavioural outputs; forward velocity (top) is the same as in **b**. Columns of each row are ordered based on the ordering of forward velocity (top). **d**, For a dynamic stimulus sequence of a fictive female only varying her size, we used our approach in **a** to identify the sufficient and necessary model LC units for the male forward velocity of the male (top). Red crosses denote inactivation; each square represents a model LC unit; colours match those in Fig. 3a. The active model LC units in the middle row are the same as those inactivated in the bottom row. **e**, Other example behavioural outputs and stimulus sequences to assess necessity and sufficiency. Same format as in **d**. For predicting Pslow song (right column), all but LC11 and LC25 were required, although not every LC type contributed as strongly. **f**, Results of cumulative inactivation for the dynamic stimulus sequences in **d**,**e**. Same format, colour legend and ordering of columns as in **c**. Source data

Given that visual features appeared to be distributed across the LC population (Figs. 2 and 3), we tested the hypothesis that combinations of LC types drive the male’s singing and pursuit of the female. We systematically inactivated model LC units in different combinations (or alone)—experiments that are not easily performed in a real flies, even with excellent genetic tools—and then examined which model LC units were necessary and sufficient to guide behaviour (Fig. 4a).

We began by testing which model LC unit, when inactivated, maintained the best performance in predicting the behaviour of control flies. In a greedy and cumulative manner, we repeatedly inactivated the model LC unit that maintained the best performance while keeping all previously chosen LCs inactivated (Fig. 4b); eventually prediction performance had to decrease because of the bottleneck imposed by the model LC units. The inactivated model LC units that led to the largest drops in performance were the strongest contributors to each behaviour (Fig. 4b, rightmost dots). Separately inactivating each model LC unit resulted in little to no drop in prediction performance (Extended Data Fig. 12).

We performed this cumulative inactivation procedure for all six behavioural outputs (Fig. 4c and Extended Data Fig. 12), and found that most model LC units contributed to multiple behavioural outputs (Fig. 4c, multiple red squares per column) and that each behavioural output was driven by multiple LC units. The 1-to-1 network enabled us to characterize the behavioural role of many previously uncharacterized LC types. It uncovered a role for LC31 in all types of song production, for LC22 in male forward velocity and Pfast song production (the song type produced when males move quickly^35^), and for LC13 in turning and the production of sine song. We also found a new role for LC10a in the production of sine song, consistent with the role of P1a neurons, whose activity directly gates LC10a activity^11^, in enabling sine song production^34^. All of these predictions can be tested in future experiments, guided by the 1-to-1 network.

As we did for examining visual stimulus encoding (Fig. 3f,g), we considered the behavioural responses to stimulus sequences in which only one parameter of female motion varied at a time. Using systematic inactivation, we again identified the model LC units that were both necessary and sufficient to produce the output of the model to these stimuli. For example, we found that when we varied female size only (Fig. 4d, top, dashed line), inactivating 10 different model LC units (Fig. 4d, middle, squares with red crosses, identified via cumulative inactivation; Methods) resulted in no change in forward velocity (Fig. 4d, middle, green trace overlays black trace). This suggests that the other model LC units (Fig. 4d, middle, squares without a red cross) were sufficient to drive behaviour. We then inactivated these ‘sufficient’ model LC units (keeping all other model LC units activated) and found a large behavioural deficit (Fig. 4d, bottom, red trace does not overlay black trace), indicating that these inactivated model LC units were also necessary. That a large number of LC types were required for behaviour remained true for other stimuli and behaviours (Fig. 4e).

Across all behavioural outputs, even for these simple stimulus sequences, we found that multiple LC units contributed to each behaviour (Fig. 4f, multiple red squares per row) and that most model LC units each contributed to multiple behaviours (Fig. 4f, multiple red squares per column). We also found consistencies between these results and prior work on specific LC types—for example, LC16, LC17, and LPLC1 contribute to the angular velocity of the male in our model (Fig. 4f, ‘vary female size’), and, if optogenetically activated, also drive turns^12,26^. The results for these simple stimuli differed slightly from those for natural courtship stimuli (for example, white squares for LC12 in Fig. 4f and red squares for LC12 in Fig. 4c), suggesting that LC contributions change with context. Overall, our results support the notion that a majority of model LC units are required for the courtship behaviour of the male.

## Distributed connections of the LC population

**Fig. 5:**
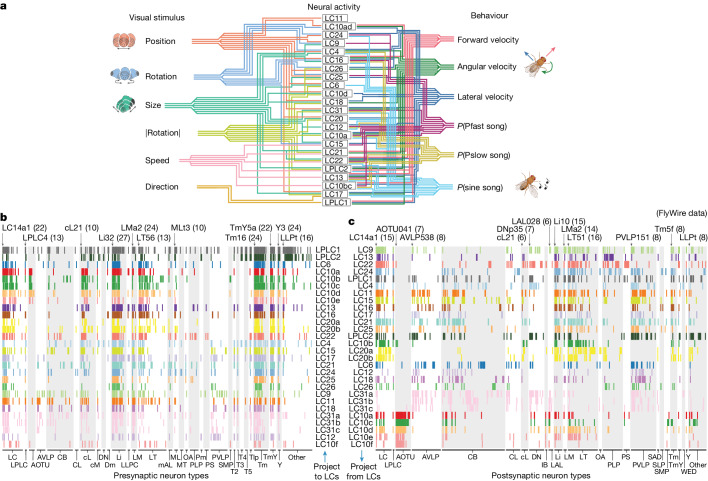
The role of LC neurons in the sensorimotor transformation of the male fly during courtship. **a**, Summary of our findings. Each line denotes a relationship between a model LC unit and a visual feature (left, *R*^2^ > 0.30 in Fig. 3b,g) or a behavioural variable (right, a normalized change in performance greater than 30% in Fig. 4c,f). A lack of connection does not rule out a relationship, as relationships may exist in other contexts or subcontexts. Even at these conservative criteria (that is, cut-offs at 0.3), many model LC units encode more than one visual feature and contribute to more than one behavioural variable. These predictions come from one training run of the 1-to-1 network; the uncertainty of each connection can be assessed by measuring differences in predictions across different training runs (Extended Data Figs. 5 and 6). **b**, Synaptic connectivity matrix for presynaptic neuron types projecting to LC or LPLC neurons. Each row is for one LC or LPLC type that we silenced in our experiments. Each column is for a presynaptic partner neuron type; columns are grouped into classes of neuron types or brain areas based on the naming conventions in the FlyWire connectome dataset^15^ (see Methods for full names) and further sorted within class such that the neuron type with connections to the largest number of LC or LPLC types is the leftmost column. A tick line indicates at least five synaptic connections were identified between neurons of an LC or LPLC neuron type and neurons of a presynaptic neuron type. We include synaptic connections for LC10a–f, LC20a–b and LC33a–c for which we have finer granularity in FlyWire than that of our genetic lines. Presynaptic neuron types with connections to large numbers of LC or LPLC neuron types are labelled—for example, Li32 (27) indicates neuron type Li32 projects to 27 out of 28 different LC neuron types considered here. Rows are sorted based on clustering LC types by their connections to presynaptic partners (Methods). **c**, Synaptic connectivity matrix for postsynaptic neuron types receiving input from LC or LPLC neurons. Same format as in **b**. We re-clustered LC types based on their connections to postsynaptic partners (rows differ from the ordering in **b**). Because this connectome dataset is from a female fruit fly, it may miss important sexually dimorphic, courtship-relevant connections to downstream areas of the male fruit fly. Source data

We aggregated results both from how the model LC neurons encode visual input (Fig. 3) and contribute to behaviour (Fig. 4) and outline these relationships with some thresholding (Fig. 5a and Methods). The picture is complicated: model LC units encode multiple visual features of the female (Fig. 5a, left connections) and contribute to multiple behavioural outputs (Fig. 5a, right connections). Even LC types involved in non-social behaviours (for example, escape), such as LC4, LC6, LPLC1 and LPLC2^21,24,26,38–40^, participate in encoding the movements of the female and driving the courtship actions of the male.

A key prediction of our 1-to-1 network is that LC neuron types share common inputs in the optic lobe (creating shared feature tuning across the LC population) and converge onto shared downstream targets to drive behaviour. To test this prediction, we analysed a recently released whole-brain connectome^15,19,41^ (FlyWire) with exhaustive cell typing in the optic lobe^18^ and central brain^19^, and in which 57 LC and LPLC neuron types have been identified so far. We computed the synaptic connectivity matrix for LC neuron types silenced in our experiments and their presynaptic cell types (Fig. 5b) as well as their postsynaptic cell types (Fig. 5c). We found that 60.2% of presynaptic neuron types projected to 2 or more LC types and 45.7% projected to 3 or more LC types (Fig. 5b, cell type Li32 projected to 27 out of 28 LC types considered). Similarly, 55.6% of downstream neurons of the same type received input from 2 or more LC types and 32.5% received input from 3 or more LC types (Fig. 5c, descending neuron type DNp35 read out from 7 out of 28 LC types considered). Thus, the LC types do share inputs and converge onto shared targets. An additional observation is that many LC and LPLC types connect directly with other LC and LPLC types in the lobula and lobula plate (Fig. 5b,c, LC and LPLC columns). Such recurrence muddles the idea that each LC type is an independent feature detector, although these lateral connections may implement a divisive normalization mechanism^42^. An important caveat is that this connectome dataset is from a female fruit fly; once the connectome of a male is generated, we can further test the predictions of the 1-to-1 network by examining putative information flow from the LCs to downstream circuits known to control chasing and singing.

## Discussion

Here we develop knockout training, a novel solution to identify a one-to-one mapping between internal units in a DNN and real neurons in the brain of a fly. The model makes predictions about how neurons respond to sensory stimuli and drive behaviour. Although silencing each LC neuron type on its own may have a small to medium effect on behaviour (Fig. 1e and Extended Data Fig. 1), our 1-to-1 network infers how the LC types work together as a population to drive the courtship behaviour of the male. We show that the model extends beyond findings from direct recordings of LC neurons^29,30^, even in behaving flies^11,31^. The 1-to-1 network provides information on LC visual responses in freely behaving flies (not head-fixed, as is required for recordings) engaging in natural social interactions and can generate LC responses to any arbitrary visual stimulus. In fact, we demonstrate that the 1-to-1 network predicts actual responses to stimuli that the model had not seen during training (for example, Fig. 2b,c). The model also makes testable predictions about which combinations of LC types are both necessary and sufficient for specific courtship behaviours (Fig. 4). A major new finding of our work is which and to what extent LC neuron types contribute to song production, an integral part of courtship guided by visual feedback^9^. Given that the same visual stimulus sequence can drive multiple LC types (Extended Data Fig. 7), this neuron-to-behaviour relationship is not readily inferred from LC recordings alone. The 1-to-1 network is the first large-scale hypothesis of how the LC types work together to encode stimuli and contribute to behaviour; we share our model and code (https://github.com/murthylab/one2one-mapping) with the community to inspire future experiments and models.

A main conclusion of this study is that the complex courtship behaviour of the male relies on combinations of visual projections neurons—including those also involved in non-social behaviours. However, we do not yet know the extent to which other behaviours beyond those observed during courtship also rely on a population code. Knockout training on the LC types could easily be applied to other visuomotor behaviours (for example, escape responses or flight) to make direct comparisons. Given the extent of interconnectivity between LC types and convergence of LC types onto common downstream cell types (Fig. 5b,c), we posit that population coding for behaviour, particularly in natural contexts, might be the norm. By contrast, for behaviours that rely on quick and robust processing, such as escape from a predator, the arrangement of LC types into optic glomeruli may facilitate the fast readout of specific channels^24^. One issue raised by the use of a multiplexed code is how the fly brain produces the correct behaviour at the correct time. For example, LPLC2 neurons synapse onto the giant fibre neuron to drive an escape take-off^25^, but our 1-to-1 network predicts that this same cell type encodes female size and contributes to the forward velocity of the male during courtship (Fig. 5a); recent work has also found LPLC2 contributes to evasive flight turns^38^. Future experiments are needed to understand how the same LC cell type can contribute to different behaviours in different contexts.

Our modelling approach comes with limitations. For example, if silencing an LC type does not lead to a noticeable change in behaviour, the 1-to-1 network cannot infer the tuning of that LC type. In addition, many silenced LC types resulted in stronger—not weaker—courtship (Fig. 1d,e), suggesting that these LC neurons may act partially as distractors to prevent relentless pursuit of the female^43,44^. We also found some mismatches between real LC responses and the responses of the 1-to-1 network (Fig. 2); although this may be owing to differences in internal state between freely moving males during natural courtship (training data for the model) versus head-fixed males passively viewing stimuli (neural recordings), training on neural data and behavioural data together may help to improve both neural and behavioural prediction (Extended Data Fig. 8). An experimental limitation of using natural behaviour arises because the statistics of the visual experience cannot be matched between LC-silenced and control males (for example, an LC9-silenced male spends much less time near the female); future experiments can use virtual reality^11^ or robotic females^44^ to present identical stimulus sequences to control and silenced males.

Following recent studies using DNNs to predict responses of visual neurons^1,3^, we used DNNs in our 1-to-1 network that are highly expressive function approximators but lack biological realism. Our model-agnostic knockout training procedure can be used to train more biologically inspired models^5,45^ that incorporate constraints from the FlyWire connectome^15,18^ and emerging male brain wiring diagrams^33^ to include recurrent connections, lateral connections between LC types (Fig. 5b,c) and delays^46^. An intriguing future direction is to apply this framework to other bottlenecks within the *Drosophila* brain, such as the descending and ascending neurons that link the brain and nerve cord^19^, and in more complex systems for which we also have genetic control over cell types^47,48^. Our work shows that constraining models with causal perturbations of neurons during complex behaviour is an important ingredient in revealing the relationships between stimulus, neurons and behaviour.

## Methods

### Flies

For all experiments, we used four- to seven-day-old virgin flies collected from density-controlled bottles seeded with eight males and eight females. Fly bottles were kept at 25 °C and 60% relative humidity. Virgin flies were housed individually and kept in behavioural incubators under a 12 h:12 h light:dark cycling; individual males were paired with a pheromone insensitive and blind (PIBL) female to encourage longer courtship sessions—see Supplementary Table 1 for more info on genotype. UAS-TNT-C was obtained from the Bloomington Stock Center. All LC split-GAL4 lines and the spGAL4 control line^33,49^ were generously provided by M. Reiser, A. Nern and G. Rubin—see Supplementary Table 2 for more information. We note that LC10 has seven different types (LC10a–g) whose genetic lines have not all been isolated; their names come from prior cell typing based on light microscopy^12^. LC10 genetic line names have not yet been mapped to these new types identified in the connectome.

### Courtship experiments

Behavioural chambers were constructed as previously described^9,50^. Each recording chamber had a floor lined with white plastic mesh and equipped with 16 microphones (Extended Data Fig. 1). Video was recorded from above the chamber at a 60 Hz frame rate; features for behavioural tracking were extracted from the video and downsampled to 30 Hz for later analysis. Audio was recorded at 10 kHz. Flies were introduced gently into the chamber using an aspirator. Recordings were timed to be within 150 min of the behavioural incubator lights switching on to catch the morning activity peak. Recordings were stopped either after 30 min or after copulation, whichever came sooner. All flies were used; we did not use any criteria (for example, if males sang during the first 5 min of the experiment or not) to drop fly sessions from analyses. In total, behaviour was recorded and analysed from 459 pairs; the number of flies per condition were as follows:

**Table:** 

LC type	LC4	LC6	LC9	LC10a	LC10ad	LC10bc	LC10d	LC11	LC12
number of pairs	17	19	18	13	15	16	16	14	14

**Table:** 

LC type	LC13	LC15	LC16	LC17	LC18	LC20	LC21	LC22	LC24
number of pairs	17	16	19	14	14	16	15	22	18

**Table:** 

LC type	LC25	LC26	LC31	LPLC1	LPLC2	control	total
number of pairs	16	18	24	16	17	75	459

Joint positions for the male and female for every frame were tracked with a DNN trained for multi-animal pose estimation called SLEAP^51^. We used the default values for the parameters and proofread the resulting tracks to correct for errors. We estimated the presence of sine, Pfast and Pslow song for every frame using a song segmenter on the audio signals recorded from the chamber’s microphones according to a previous study^35^.

From the tracked joint positions and song recordings, we extracted the following six behavioural variables of the male fly that represented his moment-to-moment behaviour. (1) ‘Forward velocity’ was the difference between the male’s current position and his position one frame in the past; this difference in position was projected onto his heading direction (that is, the vector from the male’s thorax to his head). (2) ‘Lateral velocity’ was the same difference in position as computed for forward velocity except this difference was projected onto the direction orthogonal to the male’s heading direction; rightward movements were taken as positive. (3) ‘Angular velocity’ was the angle between the male’s current heading direction and the male’s heading direction one frame in the past; rightward turns were taken as positive, and angles were reported in degrees (that is, a turn to a male’s right is 90°, a turn to his left is −90°). (4) ‘Probability of sine song’ was computed as a binary variable for each frame, where a value of 1 was reported if sine song was present during that frame, else 0 was reported. (5) ‘Probability of fast pulse (Pfast) song’ and (6) ‘probability of slow pulse (Pslow) song’ were computed in the same manner as that for the probability of sine song. These six behavioural output variables described the male’s movements (forward, lateral, and angular velocity) as well as his song production (probability of sine, Pfast and Pslow song).

Often a male fly spends periods of time without noticeable courtship of the female (for example, the ‘whatever’ state as defined in ref. ^52^). During these periods, the male probably does not rely much on the visual feedback of the female to guide his behaviour; this makes predicting his behaviour only from visual input difficult. In addition, these time periods can make up a large enough fraction of the training data to bias models to output ‘do nothing’ owing to the imbalanced training data. To mitigate these effects, we devised a set of loose criteria to identify ‘courtship frames’ in which the male is likely in a courtship state (for example, chasing or singing to the female); we then only train and test on these courtship frames.

We devised the following four criteria to determine if a frame is a courtship frame:

1. The male and female distance (taken between the joint positions of their thoraxes) averaged over the time window is less than 5 mm.
2. The proportion of frames in which the male produced song (Pfast, Pslow, or sine) during the time window is greater than 0.1.
3. The angle of the female’s location from the male’s heading direction (with respect to the male’s head), averaged over the time window, is no more than 45 visual degrees.
4. The male is traveling at least 4.5 mm/s towards the female, averaged over the time window.

The time window was 20 s long, centred on the candidate frame. Only one criterion needed to be met to classify a frame as a courtship frame. Given these criteria, roughly 70% of all frames in control sessions were considered as courtship frames. Although silencing an LC type likely alters the amount of courtship during a session, we ensured that enough courtship frames were present for training the model. LC9-silenced males had the lowest percentage of courtship frames over the entire session at 42% (consistent with its high male-to-female distance, Fig. 1e, top); the average across LC types was roughly 70% and similar to that of control sessions.

### Visual input reconstruction

To best mimic how a male fly transforms his retina’s visual input into behaviour, we desired an image-computable model (that is, one that takes as input an image rather than abstract variables determined by the experimenter, such as female size or male-to-female distance). We approximately reconstructed the male’s visual input based on pose estimation of both the male and female fly during courtship, as described in the following process. For each frame, we created a 64-pixel × 256-pixel greyscale image with a white background. Given the female rotation, size and location (see below), we placed an image patch of a greyscale fictive female (composed of ellipses that represented the head, eyes, thorax and tail of the female; no wings were included) occluding the white background. Because male flies perceive roughly 160 visual degrees on either side^53^, we removed from the image the 40 visual degrees directly behind the male, leading to images with 64 × 228 pixels. Example input images are shown in Fig. 1f, where the reconstructed female flies were coloured and on grey background for illustrative purposes. Example videos of input image sequences are present in Supplementary Videos 1 and  2.

We computed the female’s rotation, size and location in the following way. For female rotation, we computed the angle between the direction of the male head to female body and the direction of the female’s heading. A rotation angle of 0° indicates the female is facing away from the male, ±180° indicates the female is facing towards the male, and −90° or +90° indicates the female is facing to the left or right of the male, respectively. We pre-processed a set of 360 image patches (25 × 25 pixels) that depicted a rotated female for each of 360 visual degrees. Given the computed rotation angle, we accessed the image patch corresponding to that rotation angle. For female size, we treated the female fly as a sphere (whose diameter matched the average length of a female fly from head to wing tips, ~4 mm) and computed as size the visual angle between the two vectors of the male’s head position to the two outermost points on the sphere that maximize the visual angle (that is, the two furthest points along the horizontal centre line); this angle was normalized so that a size of 1 corresponded to 180 visual degrees. This size determined the width (and height, equal to the width) of the selected image patch to be placed into the 64 × 228-pixel image. Here, size indicates the size of the image patch, not the actual size of the fictive female (which may vary because a female facing away is smaller than a female facing to the left or right). For reference, for a fictive female with a size of 1.0 and facing away from the male in the centre of his visual field, her body subtends 65 visual degrees. For female position, we computed the visual angle between the male’s heading direction and the direction between the male’s head and the female’s body position. We normalized this angle such that a position of 0 is directly in front of the male, a position of either −1 or 1 is directly behind the male fly, and a position of −0.5 or +0.5 is 90 visual degrees to the left or right, respectively. We then used this position to place the image patch (with its chosen rotation and size) at a certain pixel location along the horizontal centre line of the image. Because the male and female flies did not have room to fly in the experimental chamber, we assumed that only the female’s lateral position (and not vertical position) could change.

### Description of 1-to-1 network

We designed our 1-to-1 network to predict the male fly’s behaviour (that is, movement and song production) only from his visual input. Although the male can use other sensory modalities such as olfaction or mechanosensation to detect the female, we chose to focus solely on visual inputs because: (1) the male relies primarily on his visual feedback for courtship chasing and singing^9,44^; and (2) we wanted the model to have a representation solely based on vision to match the representations of visual LC neurons.

The 1-to-1 network comprised three parts: a vision network, an LC bottleneck, and a decision network (Fig. 1a). Hyperparameters, such as the number of filters in each layer, the number of layers, and the types of layers were chosen based on prediction performance assessed on a validation set of the control sessions separate from the test set. Unless specified, each convolutional or dense layer was followed by a batchnorm operation^54^ and a relu activation function. The 1-to-1 network took as input the images of the 10 most recent time frames (corresponding to ~300 ms)—longer input sequences did not lead to an improvement in predicting behaviour. Each greyscale image was 64 × 228 pixels (with values between 0 and 255) depicting a fictive female fly on a white background (see ‘Visual input reconstruction’). Before being fed into the network, the input was first re-centred by subtracting 255 from each pixel intensity to ensure the background pixels had values of 0. The model’s output was six behavioural variables of the male fly: forward velocity, lateral velocity, angular velocity, probability of sine song, probability of Pfast song, and probability of Pslow song (see ‘Courtship experiments’).

#### Vision network

The first layer of the vision network was spatial convolutions with 32 filters (kernel size 3 × 3) and a downsampling stride of 2. The second and third layers were identical to the first except with separable 2D convolutions^55^. The final layer was a two-stage linear mapping^56^ which first spatially pools its input of activity maps and then linearly combines the pooled outputs across channels into 16 embedding variables; pooling the spatial inputs in this manner greatly reduced the number of parameters for this layer. Batchnorm and relus did not follow this two-stage layer. The vision network processed each of the 10 input images separately; in other words, the vision network’s weights were shared across time frames (that is, a 1D convolution in time). Allowing for 3D convolutions of the visual inputs (that is, 3D kernels for the two spatial dimensions and the third time dimension) did not improve prediction performance (Extended Data Fig. 3), likely because of the increase in the number of parameters. For simplicity, the vision network’s input was the entire image (that is, the entire visual field); we did not include two retinae. We found that incorporating two retinae into the model, while more biologically plausible, made it more difficult to interpret the tuning of each LC neuron type. For example, for a two-retinae model, it is difficult to determine if differences in tuning for two model units of the same LC type but in different retinae are true differences in real LC types or instead differences due to overfitting between the two retinal vision networks. The 1-to-1 network avoids this discrepancy through the simplifying assumption that each LC type has a similar response across both retinae.

#### LC bottleneck

The next component of the DNN model was the LC bottleneck, which received 10 16-dimensional embedding vectors corresponding to the past 10 time frames. These embedding vectors were passed through a dense layer with 64 filters followed by another dense layer with number of filters equal to the number of silenced LC types (23 in total). We call the 23-dimensional output of this layer the ‘LC bottleneck’. Each model LC unit represents the summed activity of all neurons of the same LC type (that is, projecting to the same optic glomerulus), which makes it easy to compare to calcium imaging recordings of LC neurons which track the overall activity level of a single glomerulus. We found that adding additional unperturbed ‘slack’ model LC units to match the total number of LC types (for example, 45 model LC units instead of 23 units) did not improve prediction performance; in the extreme case, adding a large number slack variables encourages the network to ignore the ‘unreliable’ knocked-out units in favor of predicting shared behaviour across silenced and control sessions (that is, similar to training without knockout). For two perturbations (LC10ad and LC10bc), the genetic lines silenced two LC neuron types together. For simplicity, we assigned each of these to its own model LC unit, which represented the summed activity of all neurons from both types (for example, LC10a and LC10d for LC10ad). Because the LC bottleneck reads from all 10 past time frames, each model LC unit integrates information over time (for example, for motion detection). Additionally, the model LC responses are guaranteed to be nonnegative because of the relu activation functions.

#### Decision network

The decision network took as input the activations of the 23 LC bottleneck units and comprised 3 dense layers, where each layer had 128 filters. The decision network predicted the movement output variables (forward velocity, lateral velocity, and angular velocity) each with a linear mapping and the song production variables (probability of sine, Pfast and Pslow song) each with a linear mapping followed by a sigmoid activation function.

### Knockout training

We sought a one-to-one mapping between the model’s 23 LC units in its bottleneck and the 23 LC neuron types in our silencing experiments (Fig. 1a). To identify this mapping, we devised knockout training. We first describe the high-level training procedure and then give details about the optimization. For a randomly initialized 1-to-1 network, we arbitrarily assigned model LC units to real LC types (that is, in numerical order). For each training sample, we knocked out (that is, set to 0 via a mask) the model LC unit that corresponded to the silenced LC type; no model units were silenced for control data (Fig. 1b). This is similar to dropout training^36^ except that hidden units were purposefully—not randomly—chosen. The intuition behind knockout training is that the remaining unperturbed model LC units must encode enough information or ‘pick up the slack’ to predict the silenced behaviour; any extra information will not be encoded in the unperturbed units (as the back-propagated error would not contain this information). For example, let us assume that female size is encoded solely by LPLC1 and that this cell type contributes strongly to forward velocity. To predict the forward velocity of LPLC1-silenced males (which would not rely on female size), the other model LC units would need only to encode other features of the fictive female (for example, her position or rotation). In fact, any other model LC unit encoding female size would hurt prediction because forward velocity of LPLC1-silenced males does not depend on it. Another view of knockout training is that we optimize the model to predict behaviour while also constraining the model on which internal representations it may use. These constraints are set by the perturbations (for example, genetic silencing) we use in our experiments.

The optimization details are as follows. The model was trained end-to-end using stochastic gradient descent with learning rate 10^−3^ and momentum 0.7. Each training batch had 288 samples, where each sample was a sequence of 10 images and 6 output values. Each batch was balanced across LC types (24 in total including control), where each LC type had 12 samples. The batch was also balanced for types of song (sine song, pulse song, or no song), as different flies sang different amounts of song. The model treated different flies for the same silenced LC type as the same to capture overall trends of an ‘average’ silenced fly. We *z*-scored the movement behavioural variables (forward, lateral, and angular velocity) based on the mean and standard deviation of the control data in order to have similarly sized gradients from each output variable. The loss functions were mean squared error for forward, lateral, and angular velocity and binary cross-entropy for the probabilities of sine, Pfast, and Pslow song. The model instantiation and optimization was coded in Keras (https://keras.io/) on top of Tensorflow^57^; we used the default random initialization parameters to initialize weights. We stopped training when prediction performance for forward velocity (evaluated on a validation set, see below) began to decrease (that is, early stopping).

#### Training and test data

After identifying courtship frames (see ‘Courtship experiments’), we split these frames into train, validation and test sets. To form a test set for a given LC type (or control), we randomly selected 3-s windows across all flies until we had 15 min of data (27,000 frames). Selecting windows instead of randomly choosing time frames ensured that no frame in the visual input of the test data overlapped with any training frames. For control sessions, after selecting the test set, we also randomly sampled from the remaining frames to form a validation set (27,000 frames) in the same way as we did for the test set; the validation set was used for hyperparameter choices and early stopping. All remaining frames were used for training. To balance the number of frames for each LC type and control, we randomly sampled at most 600,000 frames (~5.5 h) across sessions for each LC type and control. This ensured no single LC type or control was over-represented in the training data (that is, a class imbalance). In total, our training set had ~11.6 million training samples. To account for the observation that flies tend to prefer to walk along the edge of the chamber in either a clockwise or counter-clockwise manner—biasing lateral and angular velocities to one direction—we augmented the training set by flipping the visual input from left to right and correspondingly changing the sign of the lateral and angular velocities; each training sample had a random 50% chance of being flipped. No validation or test data were augmented.

#### Dropout and no knockout training

**Extended Data Fig. 4:**
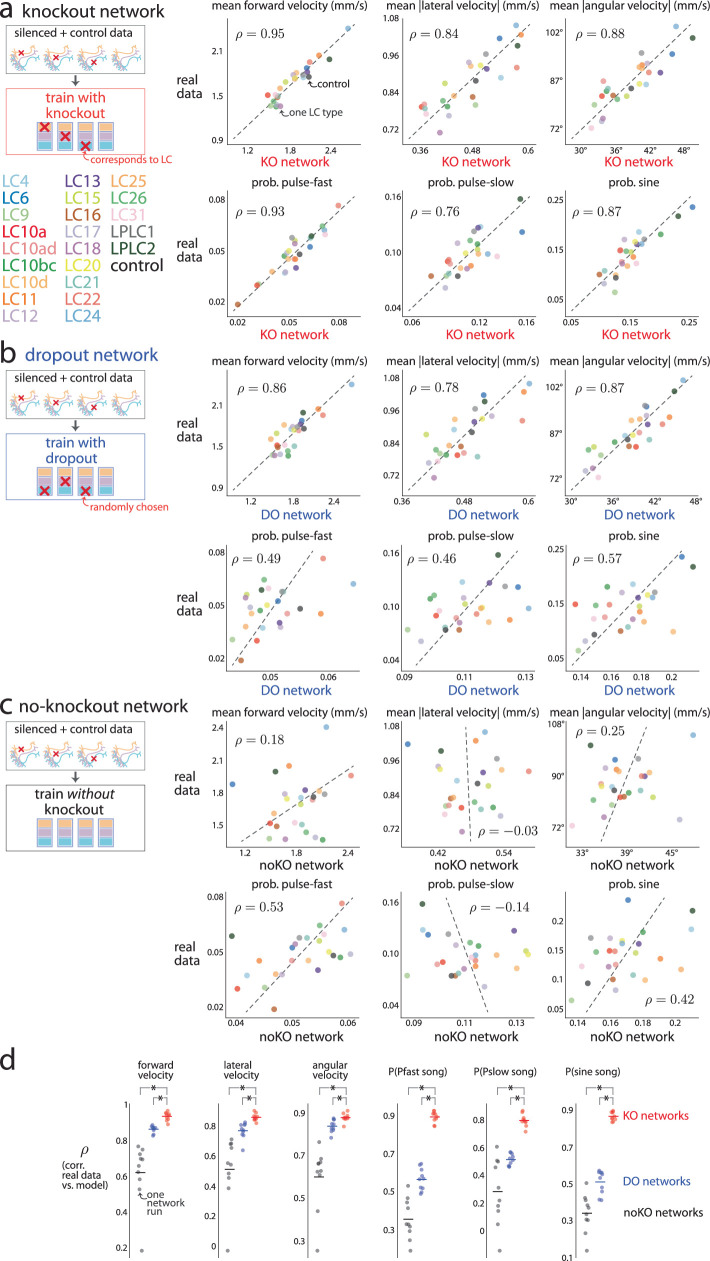
Assessing model predictions of mean changes in behavior. Given the mean changes in behavior due to silencing (where the mean is taken over the entire session, Extended Data Fig. 1), we wondered to what extent the knockout (KO) network predicted these overall changes versus training a dropout (DO) network, for which a randomly-chosen model LC unit was inactivated during training, and a noKO network, for which no inactivation of any model LC unit was performed during training. **a**. For each LC neuron type, we computed the average behavior across all held-out courtship frames in the test set (‘real data’). We then computed the mean behavior as predicted by the KO network across the same frames (‘KO network’). Each dot denotes one LC type (color dots) or control session (black dot); colors are indicated at left. Dashed lines are the best linear fit; the correlation *ρ* is taken across all LC types excluding the control sessions. The KO network has large *ρ*’s across behavior outputs, indicating good prediction of overall changes. **b**. Same as in **a** except for the DO network; for evaluation, no model LC units were inactivated (i.e., dropout was used for regularization^36^). Correlations were smaller for the DO network than for the KO network (compare *ρ*’s between **a** and **b**). However, for the movement variables, correlations for the DO network were only slightly smaller than those of the KO network. Because the DO network had no access to which LC type was silenced, this suggests that the statistics of visual inputs differed across LC types. For example, imagine if the DO network accurately predicted the behavior of control male flies, including that the male does not sing when the female is far away. Then, if silencing LC10a resulted in the male not being interested in courting the female, the female would be far away in most frames, and the DO network would correctly predict a decrease in song production, even though the DO network has no knowledge LC10a was silenced. Thus, DO training is an appropriate control to ask whether the sensorimotor transformation has changed or if the male has altered his desire to pursue courtship. This also motivates future experiments with virtual reality where the male’s visual statistics can be matched between LC-silenced and control males. **c**. Same as in **a **and **b **except for the noKO network. Correlations were substantially smaller than for the KO and DO networks (compare *ρ*’s between **a **and **c**), indicating that the noKO network could not recover behavior from LC-silenced flies. **d**. We trained 10 networks each for KO, DO, and noKO training. Each of the 10 networks had different random initializations and different random orderings of training samples. For a fair comparison, the same initialized network and ordering was shared across KO, DO, and noKO training for each of the 10 runs. We then computed the *ρ*’s of overall mean behaviors for each network and real data. For each of the six behavioral outputs, we found that the KO network predicted the changes in behavior across LC types better than the predicted changes for the DO and noKO networks (red dots above blue and black dots). Each dot denotes one network, and each asterisk denotes that the mean of the KO network is significantly greater than the mean of either the DO or noKO network (*p* < 0.05, paired, one-tailed permutation test, *n* = 10). Network run 1 was chosen as the exemplar network in **a**-**c **(as well as in Figs. 1–4). Source data

For comparison to knockout (KO) training, we considered three networks with the same architecture as the 1-to-1 network but trained with other procedures (Extended Data Fig. 3). First is the untrained network for which no training is performed (that is, all parameters remain at their randomized initial values). Second, we performed a version of dropout (DO) training^36^ by setting to 0 a randomly chosen model LC unit for each training sample independent of the sample’s silenced LC type; no model LC unit’s values are set to 0 for samples from control sessions. This training procedure knocks out the exact same number of units as that of knockout training. No dropout is performed during inference. Third, we consider training a network without knocking out (noKO) any model LC units. We trained the DO and noKO networks with the exact same data as that for KO training (a combined dataset of courtship sessions from 23 different LC types and control), but the DO and noKO networks were not given any information about which LC type was silenced for a training sample. This makes the DO and noKO fair null hypotheses: The DO and noKO networks assume that no change in behaviour occurs between LC-silenced males and control males, whereas the KO network attempts to find these differences. The DO and noKO networks helped us to ground the prediction performance of knockout training when predicting moment-to-moment behaviour (Extended Data Figs. 3 and 4) and real LC responses (Fig. 2e) as well as consistency in training (below).

#### Consistency across different training runs

**Extended Data Fig. 5:**
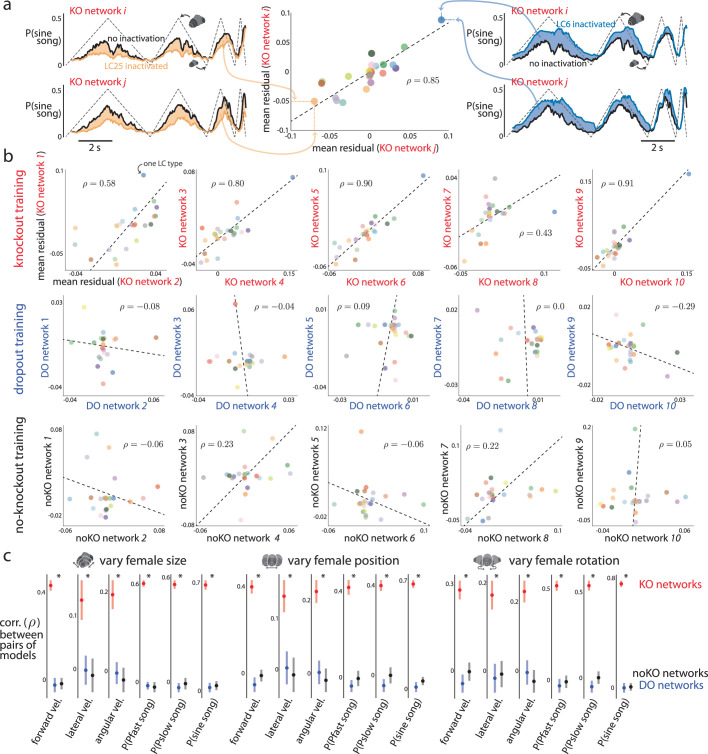
Consistency in behavioral predictions across networks with different random initializations. Deep neural networks with the same architecture and trained on the same data may converge to different internal representations depending on their parameter initializations and the ordering of training samples observed by stochastic gradient descent. We wondered to what extent the solution identified by knockout (KO) training changes for different random initializations and different orderings of training data. If KO training is consistent for the 1-to-1 network’s architecture, then we would expect to see that different training runs of a KO network should converge to similar predictions in behavior. See Extended Data Fig. 6 for a similar analysis of the 1-to-1 network’s consistency in neural predictions. **a**. To test this, we trained 10 KO networks, each with a different random initialization and different ordering of training samples. We then passed as input a dynamic stimulus sequence in which the fictive female varied her size over time (dashed trace in top left plot; female position and rotation remained fixed). Inactivating LC25 (orange line, top left plot) resulted in an overall decrease in the probability of song relative to that of no inactivation (black line, top left plot); we can compute the overall change in behavior by taking the mean residual between the two (orange shade, top left plot). If two KO networks were consistent, we would expect that this KO network *i* should match its mean residual if we were to perform the same procedure for another KO network *j*; indeed, this is what we saw (compare top and bottom panels on the left). Inactivating LC6 resulted in an increase in probability of song for both KO networks (rightmost plots). We quantified the consistency between the two KO networks by computing the correlation across LC types of the time-averaged residuals (middle scatter plot); a correlation *ρ* close to 1 indicates that both KO networks consistently have the same predictions of behavior for different silenced LC types. **b**. Scatter plots for 5 pairings of the 10 KO networks (top row) of the time-averaged residuals of probability of sine song for the stimulus in which the fictive female varies in size (same as in **a**). Each dot denotes one LC type, and colors correspond to LC names in Extended Data Fig. 4. Dashed lines denote best linear fit. We also assessed the consistency of DO networks (middle row) and noKO networks (bottom row), which were substantially lower than the *ρ*’s for the KO network. **c**. Correlations of time-averaged residuals for the three dynamic stimulus sequences and all six behavioral outputs. Each dot is the mean across all 45 pairs of networks; error bars denote 1 s.e.m. The KO networks had significantly larger mean pairwise correlations (asterisk denotes *p* < 0.05, paired permutation test, *n* = 45) than those for the DO and noKO networks (red dots above blue and black dots) for all stimulus sequences and behavioral outputs. We conclude that the KO networks are consistent in behavioral predictions. An important use of the ensemble of 10 KO networks is for estimating model uncertainty for a particular stimulus sequence. A single KO network can only give one prediction for a stimulus sequence (Fig. 4d,e); one may erroneously conclude that the model is equally certain about all stimulus sequences. Instead, the 1-to-1 network may be more uncertain for different stimulus sequences, especially those that are rarely observed during natural courtship. Thus, before experimentally testing the 1-to-1 network’s predictions, one may first check if the 1-to-1 network is confident in its prediction by assessing the extent to which different network runs agree on the same prediction. If there is large agreement (as seen here), the 1-to-1 network is confident in its predictions. On the other hand, a mismatch in its predictions and experimental data is more interesting than a stimulus sequence for which the 1-to-1 network is uncertain (and thus expected to not agree with experimental data). Source data

**Extended Data Fig. 6:**
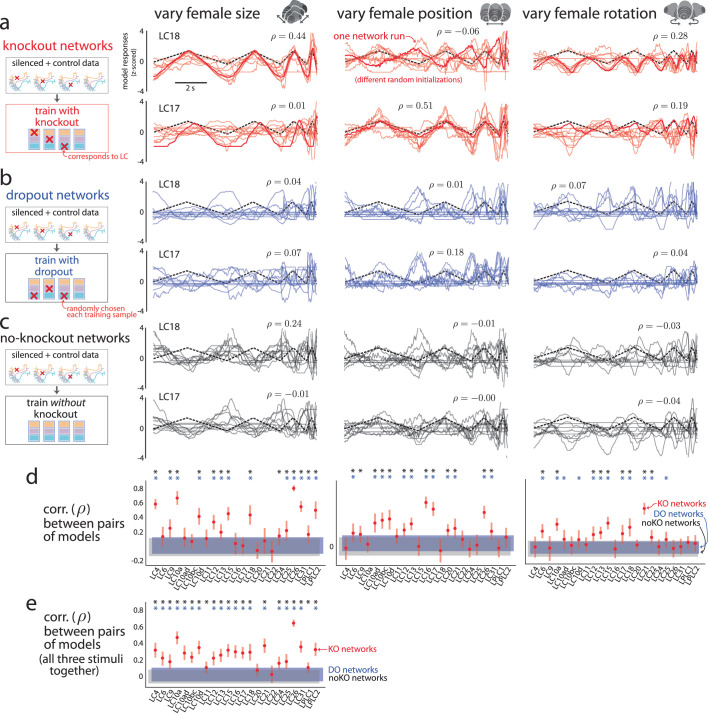
Consistency in LC response predictions across networks with different random initializations. We wondered to what extent knockout training converged to different solutions in predicting LC responses given different random initializations and different orderings of training data. See Extended Data Fig. 5 for consistency in behavioral predictions. **a**. We performed knockout training on 10 different runs—each run had a different random initialization and different random ordering of training data. We then fed into the KO networks as input three dynamic stimulus sequences in which the fictive female varied her size (left column), position (middle column), and rotation (right column) (same sequences as in Figs. 3f and 4d,e). For LC18 (top row), model responses were consistent for female size and rotation but not position. Each trace is from one KO network run; the bold trace is for network run 1 (chosen as the 1-to-1 network in Figs. 1–4). Traces across all three stimulus sequences were z-scored and then flipped in sign to ensure the largest possible mean correlation *ρ* over time (as sign is not identifiable via knockout training). For LC17 (bottom row), model responses were consistent for female position but not size or rotation, suggesting consistency was stimulus dependent. This is in line with the idea that knockout training can only identify a one-to-one mapping for stimulus sequences that lead to noticeable changes in behavior from LC-silencing (Extended Data Fig. 2); KO networks disagree on stimulus sequences that lead to little to no change in behavior, as some change is needed in order to identify an LC type’s role in driving behavior. **b**-**c**. We assessed the consistency of the dropout (DO) networks for which a randomly-chosen model LC unit was inactivated (**b**) and noKO networks for which no inactivation was performed (**c**). Both DO and noKO networks had poor consistency for LC18 and LC17 across all stimulus sequences (largest *ρ* = 0.24). **d**. We computed the mean correlation (dots) across all 45 pairs of networks and found that the KO networks had significantly larger mean correlations than DO networks (blue asterisks, *p* < 0.05, paired, one-sided permutation test, *n* = 45) and noKO networks (black asterisks, *p* < 0.05, paired, one-sided permutation test, *n* = 45) for the three different stimulus sequences. **e**. We concatenated the responses for each network across all three stimulus sequences and re-computed the mean correlation (dots). Almost all of the LC types show a significant increase in mean correlation for KO network runs versus DO network runs (blue asterisks, *p* < 0.05, paired, one-sided permutation test, *n* = 45) and noKO network runs (black asterisks, *p* < 0.05, paired, one-sided permutation test, *n* = 45). Error bars in **d **and **e **denote 1 s.e.m. Taken together, these results indicate that knockout training identified consistent KO networks that reliably predict neural responses. That KO networks were more consistent than DO and noKO networks suggests that knockout training captured meaningful changes in behavior. Because KO networks may disagree more for different stimulus sequences (a notion of uncertainty), future experiments should take this uncertainty into account when testing the 1-to-1 network’s predictions. In fact, presenting stimulus sequences for which the KO networks disagree the most may be the most informative, as we can use the responses to these sequences to rule out some of the KO networks. Source data

Because DNNs are optimized via stochastic gradient descent, the training procedure of a DNN is not deterministic; different random initializations and different orderings of the training data may lead to DNNs with different prediction performances. To assess whether the 1-to-1 network is consistent across training runs, we trained 10 runs of the 1-to-1 network with different random initializations and different random orderings of training samples. For comparison, we also trained 10 networks either with dropout training or without knockout training (above) as well as 10 untrained networks. For a fair comparison across training procedures (knockout, dropout, without knockout and untrained), each run had the same parameter initialization and ordering of training samples. We compared the 1-to-1 network to these three networks by assessing prediction performance of moment-to-moment behaviour (Extended Data Fig. 3), overall mean changes to behaviour across silenced LC types (Extended Data Fig. 4), consistency both in behavioural predictions (Extended Data Fig. 5) and neural predictions (Extended Data Fig. 6), prediction performance of real LC responses for a one-to-one mapping (Fig. 2e and Extended Data Fig. 8) and prediction performance of real LC responses for a fitted linear mapping (Extended Data Fig. 8). We opted to investigate the inner workings of a single 1-to-1 network in Figs. 3 and 4 both for simplicity and because some analyses can only be performed on a single network (for example, the cumulative ablation experiments in Fig. 4). Different runs of the 1-to-1 networks had some differences in their predictions (Extended Data Figs. 5 and 6), but the overall conclusion that the LC bottleneck in the 1-to-1 network revealed a combinatorial requirement for multiple LC types to drive the male’s courtship behaviours remained true over all runs. For our analyses in Figs. 3 and 4, we chose the 1-to-1 network that had the best prediction for both behaviour and neural responses (model 1 in Extended Data Fig. 3, and in Extended Data Fig. 8).

### Two-photon calcium imaging

We recorded LC responses of a head-fixed male fly using a custom-built two-photon microscope with a 40× objective and a two-photon laser (Coherent) tuned to 920 nm for imaging of GCaMP6f. A 562 nm dichroic split the emission light into red and green channels, which were then passed through a red 545–604 nm and green 485–555 nm bandpass filter, respectively. We recorded the imaging data from the green channel with a single plane at 50 Hz. Before head fixation, the male’s cuticle above the brain was surgically removed, and the brain was perfused with an extracellular saline composition. The male’s temperature was controlled at 30 °C by flowing saline through a Peltier device and measured via a water bath with a thermistor (BioscienceTools TC2-80-150). We targeted LC neuron types LC6, LC11, LC12, LC15 and LC17 (Fig. 2a) for their proximity to the surface (and thus better imaging signal), prior knowledge about their responses from previous studies^29–31^, and because they showed changes to male behaviour when silenced (Fig. 1e and Extended Data Fig. 1).

Each head-fixed male fly walked on an air-supported ball and viewed a translucent projection screen placed in the right visual hemifield (matching our recording location in the right hemisphere). The flat screen was slanted 40 visual degrees from the heading direction of the fly and perpendicular to the axis along the direction between the fly’s head and the centre of the screen (with a distance of 9 cm between the 2). An LED-projector (DLP Lightcrafter LC3000-G2-PRO) with a Semrock FF01-468/SP-25-STR filter projected stimulus sequences onto the back of the screen at a frame rate of 180 fps. A neutral density filter of optical density 1.3 was added to the output of the projector to reduce light intensity. The stimulus sequences (described below) comprised a moving spot and a fictive female that varied her size, position and rotation.

We recorded a number of sessions for each targeted LC type: LC6 (5 flies), LC11 (5 flies), LC12 (6 flies), LC15 (4 flies) and LC17 (5 flies). We imaged each glomerulus at the broadest cross-section, typically at the midpoint, given that we positioned the head of the fly to be flat (tilted down 90°, with the eyes pointing down). We hand selected regions of interest (ROIs) that encompassed the shape of the glomerulus within the 2D cross-section. We computed Δ*F*/*F*_0_ for these targeted ROIs using a baseline ROI for *F*_0_ that had no discernible response and was far from targeted ROIs. For each LC and stimulus sequence, we concatenated repeats across flies. To remove effects due to adaptation across repeats and differences among flies, we de-trended responses by taking the *z*-score across time for each repeat; we then scaled and re-centred each repeat’s *z*-scored trace by the standard deviation and mean of the response trace averaged across all the original repeats (that is, the original and denoised repeat-averaged trace had the same overall mean and standard deviation over time). To test whether an LC was responsive to a stimulus sequence or not, we computed a metric akin to a signal-to-noise ratio for each combination of LC type and stimulus sequence in the following way. For a single run, we split the repeats into two separate groups (same number of repeats per group) and computed the repeat-averaged response for each group. We then computed the *R*^2^ between the two repeat-averaged responses by computing the Pearson correlation over time and squaring it. We performed 50 runs with random split groups of repeats to establish a distribution of *R*^2^ values. We compared this distribution to a null distribution of *R*^2^ values that retained the timecourses of the responses but none of the time-varying relationships among repeats. To compute this null distribution, we sampled 50 runs of split groups (same number of repeats as the actual split groups) from the set of repeats for all stimulus sequences; in addition, the responses for each repeat were randomly reversed in time or flipped in sign, breaking any possible co-variation across time among repeats. For each combination of LC type and stimulus, we computed the sensitivity^58^ *d*′ between the actual *R*^2^ distribution and the null *R*^2^ distribution. We designated a threshold *d*′ > 1 to indicate that an LC was responsive for a given stimulus sequence (that is, we had a reliable estimate of the repeat-averaged response). After this procedure, a total of 27 combinations of stimulus sequence and LC type out of a possible 45 combinations remained (Extended Data Fig. 8).

We considered two types of stimulus sequences: a moving spot and a moving fictive female. The moving spot (black on isoluminant grey background) had three different stimulus sequences (Fig. 2b,c). The first stimulus sequence was a black spot with fixed diameter of 20° that moved from the left to right with a velocity chosen from candidate velocities {1, 2, 5, 10, 20, 40, 80} ° s^−1^; each sequence lasted 2 s. The second stimulus sequence was a spot that loomed from a starting diameter of 80° to a final diameter of 180° according to the formula $\theta (t)=-2{\tan }^{-1}(-r/v\cdot 1/t)$, where *r*/*v* is the radius-to-speed ratio with units in ms and *t* is the time (in ms) until the object reaches its maximum diameter^21^ (that is, *t* = *t*_final_ − *t*_current_). A larger *r*/*v* corresponds with a slower object loom. We presented different loom speed ratios chosen from candidate *r*/*v* ∈ {10, 20, 40, 80} ms. Once a diameter of 180° was reached, the diameter remained constant. The third stimulus sequence was a spot that linearly increased its size from a starting diameter of 10° according to the formula *θ* = 10 + *v* ⋅ *t*, where *v* is the angular velocity (in ° s^−1^) and *t* is the time from stimulus onset (in seconds). The final diameter of the enlarging spot for each velocity (30°, 50°, 90° or 90°, respectively) was determined based on the chosen angular velocity *v* ∈ {10, 20, 40, 80} ° s^−1^. Once a diameter of 90° was reached, the diameter remained constant.

The second type of stimulus sequence was a fictive female varying her size, position, and rotation. The fictive female was generated in the same manner as that for the input of the 1-to-1 network (see ‘Visual input reconstruction’). We took the angular size of the fictive female (65 visual degrees for a size of 1.0, where the female faces away from the male at the centre of the image) and used it to set the angular size of the fictive female on the projection screen. We considered three kinds of fictive female stimulus sequences with 9 different sequences in total (Supplementary Video 1 and Extended Data Fig. 8); we first describe them at a high level and then separately in more detail. The first kind consisted of sequences in which the female varied only one visual parameter (for example, size) while the other two parameters remained fixed (for example, position and rotation); we varied this parameter with three different speeds. Second, we generated sequences that optimized a model output variable (for example, maximizing or minimizing forward velocity). Third, we used a natural image sequence taken from a courtship session. Each stimulus sequence lasted for 10 s (300 frames).

Details of the fictive female sequences are as follows. For reference, a size of 1.0 is ~65 visual degrees, and a position of 0.5 is 90 visual degrees to the right from centre.

- Vary female position: the female varied only her lateral position (with a fixed size of 0.8 and a rotation angle of 0° facing away from the male) from left to right (75 frames) then right to left (75 frames). Positions were linearly sampled in equal intervals between the range of −0.1 and 0.5. This range of positions was biased to the right side of the visual field to account for the fact that the projection screen was oriented in the male’s right visual hemifield. After the initial pass of left to right and right to left (150 frames total), we repeated this same pass two more times with shorter periods (100 frames and 50 frames in total, respectively), interpolating positions in the same manner as the initial pass.
- Vary female size: the same generation procedure as for ‘vary female position’ except that instead of position, we varied female size from 0.4 to 0.9 (sampled in equal intervals) with a fixed position of 0.25 and a rotation angle of 0° facing away from the male.
- Vary female rotation: the same generation procedure as for ‘vary female position’ except that instead of position, we varied the female rotation angle from −180° to 180° (sampled in equal intervals) with a fixed position of 0.25 and a fixed size of 0.8.
- Optimize for forward velocity: we optimized a 10-s stimulus sequence in which female size, position, and rotation were chosen to maximize the 1-to-1 network’s output of forward velocity for 5 s and then minimize forward velocity for 5 s. In a greedy manner, the next image in the sequence was chosen from candidate images to maximize the objective. We confirmed that this approach did yield large variations in the model’s output. To ensure smooth transitions, the candidate images were images ‘nearby’ in parameter space (that is, if the current size was 0.8, we would only consider candidate images with sizes in the range of 0.75 to 0.85). Images were not allowed to be the same in consecutive frames and had to have a female size greater than 0.3 and a female position between −0.1 and 0.5.
- Optimize for lateral velocity: the same generation procedure as for ‘Optimize for forward velocity’ except that we optimized for the model output of lateral velocity. In this case, maximizing or minimizing lateral velocity is akin to asking the model to output the action of moving to the right or left.
- Optimize for angular velocity: the same generation procedure as for ‘Optimize for forward velocity’ except that we optimized for the model output of angular velocity. In this case, maximizing or minimizing angular velocity is akin to asking the model to output the action of turning to the right or left.
- Optimize for forward velocity with fixed position: the same generation procedure as for ‘Optimize for forward velocity’ except that we limited female position *p* to be within the tight range of 0.225 < *p* < 0.275. This ensured that most changes of the female stemmed from changes in either female size or rotation, not position.
- Optimize for lateral velocity with multiple transitions: the same generation procedure as for ‘Optimize for lateral velocity’ except that we had four optimization periods: maximize for 2.5 s, minimize for 2.5 s, maximize for 2.5 s and minimize for 2.5 s.
- Natural stimulus sequence: a 10-s stimulus sequence taken from a real courtship session. This sequence was chosen to ensure large variation in the visual parameters and that the female fly was mostly in the right visual field between positions −0.1 and 0.5.

For each recording session, we presented the stimuli in the following way. For the moving spot stimuli, each stimulus sequence was preceded by 400 ms of a blank, isoluminant grey screen. For the fictive female stimuli, a stimulus sequence of the same kind (for example, ‘Vary female size’) was presented in three consecutive repeats for a total of 30 s; this stimulus block was preceded by 400 ms of a blank, isoluminant grey screen. All stimulus sequences (both moving spot and the fictive female) were presented one time each in a random ordering. Another round (with the same ordering) was presented if time allowed; usually, we presented 3 to 4 stimulus rounds before an experiment concluded. This typically provided 9 or more repeats per stimulus sequence per fly.

### Predicting real neural responses

To obtain the model predictions for the artificial moving spot stimuli (Fig. 2b,c), we generated a fictive female facing away from the male and whose size and position matched that of the moving spot. This was done to prevent any artifacts from presenting a stimulus (for example, a high-contrast moving spot) on which the model had not been trained, as the model only observed a fictive female. We matched the angular size of the fictive female to that of the presented stimulus by using the measured conversion factor of 65 visual degrees for a fictive female size of 1.0. For the stimulus of the moving spot with varying speed (Fig. 2b), the fictive female translated from left to right (that is, same as the stimuli presented to the male fly). Because the 1-to-1 network’s responses could remain constant and not return to 0 for different static stimuli (that is, no adaptation mechanism), we added a simple adaptation mechanism to the model’s responses such that if responses were the same for consecutive frames, the second frame’s response would return to its initial baseline response with a decay rate of 0.1. To obtain model predictions for the fictive female stimuli (Fig. 2d,e), we input the same stimulus sequences presented to the fly except that we changed the greyscale background to white (to match the training images).

To evaluate the extent to which the 1-to-1 network predicted the repeat-averaged LC responses for each stimulus sequence of the moving fictive female, we sought an *R*^2^ prediction performance metric that accounted for the fact that our estimates of the repeat-averaged responses were noisy. Any metric not accounting for this repeat noise would undervalue the true prediction performance (that is, the prediction performance between a model and a repeat-averaged response with an infinite number of repeats). To measure prediction performance, we chose a noise-corrected *R*^2^ metric recently proposed^59^ that precisely accounts for noise across repeats and corrects for bias in estimating the ground truth normalized *R*^2^. A noise-corrected *R*^2^ = 1 indicates that our model perfectly predicts the ground truth repeat-averaged responses up to the amount of noise across repeats. We note that our noise-corrected *R*^2^ metric accounts for differences in mean, standard deviation, and sign between model and real responses, as these differences do not represent the information content of the responses.

**Extended Data Fig. 7:**
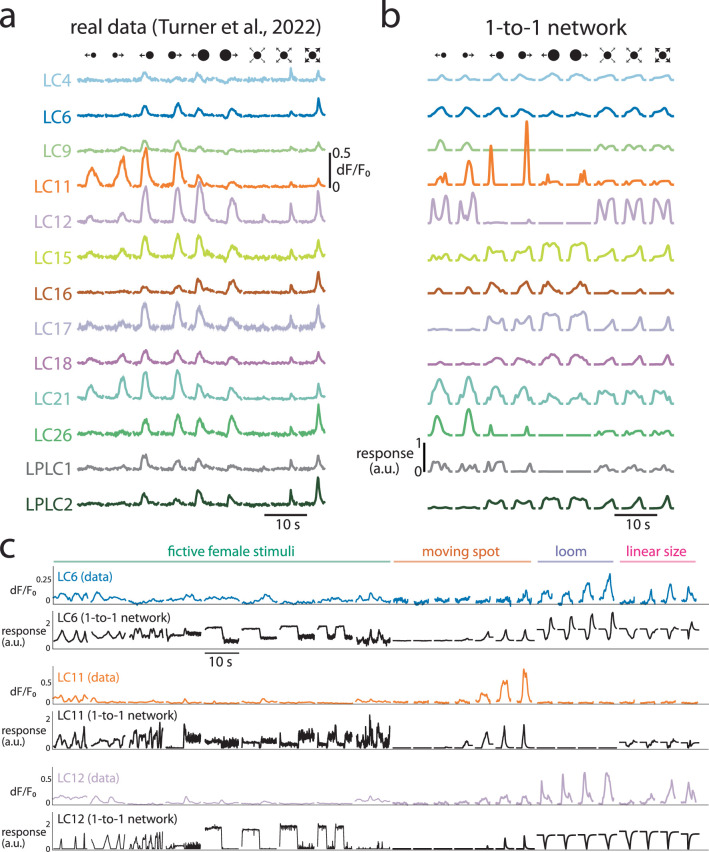
Predicting real LC responses to artificial stimuli and predicting response magnitude. **a**. In addition to our own recordings, we further tested the 1-to-1 network’s neural predictions on a large number of LC neuron types whose responses were recorded in another study^31^. One caveat was that these responses were recorded from females, not males. We considered responses to artificial stimuli of laterally moving spots with different diameters and different movement directions as well as looming spots with different loom accelerations (top row). Traces denote responses averaged over repeats and flies, shaded regions denote 1 s.e.m. (some regions are small enough to be hidden by the mean traces). Data same as in Fig. 3**a **of ref. ^31^. **b**. Model LC responses from the 1-to-1 network. We fed as input the same stimuli but changed the spot to a fictive female facing forward (to better match these artificial stimuli to the fictive female stimuli on which the 1-to-1 network was trained). For visual comparison, we matched the mean and standard deviation (taken across all stimuli) of each LC type’s model responses to those of the real LC responses; we also flipped the sign of a model LC unit’s responses to ensure a positive correlation with the real LC type (flipping was only performed for LC6 and LC21). To account for adaptation effects, model LC unit’s responses decayed to their initial baseline after no change in the original responses occurred (see Methods). Overall, it appeared that almost all the real LC neurons and model LC units respond to these artificial stimuli. Some of the best qualitative matches were LC11—where the 1-to-1 network correctly identified the object size selectivity of LC11 neurons^27^—LC15, LC17, and LC21. A failure of the model was predicting LC12 responses; this was true of our LC recordings as well (**c** and Extended Data Fig. 8). This failure may be due to an unlucky random initialization, as networks trained with knockout over 10 training runs were not in strong agreement of LC12’s responses (Extended Data Fig. 6). Another explanation is that LC12 only weakly contributes to behavior for these simplified stimuli. If this were the case, then KO training would not be able to identify LC12’s contributions to behavior nor its neural activity. One piece of evidence that this might be the explanation is that solely inactivating LC12 for simple, dynamic stimulus sequences did not lead to any change in the model’s behavioral output (Fig. 4f). For natural stimulus sequences, LC12 does appear to play a role (Fig. 4c), motivating the use of more naturalistic stimuli when recording from LC types (Fig. 2). **c**. We continued to test the 1-to-1 network’s neural predictions by comparing the model’s response magnitudes for different types of stimuli. We wondered whether the relative magnitudes of model LC responses across all stimulus sequences qualitatively matched that of real LC responses. If so, it indicates that the model’s selectivity for certain stimuli matches real LC selectivity. This is different from our quantitative comparisons that normalized model LC responses for each stimulus separately (Fig. 2d,e and Extended Data Fig. 8). We note that a priori, we would not expect the 1-to-1 network to predict response magnitude, as downstream weights could re-scale any activity of the model LC units. However, as found when comparing the internal representations of deep neural networks to one another^60^, the relative magnitudes of internal units may be an important part of encoding informative representations. Same format as in Fig. 2f for the three remaining recorded LC types (LC6, LC11, and LC12). For LC6, the 1-to-1 network correctly predicts a larger response to loom than responses to a moving spot and a spot varying its size linearly (‘linear size’); however, it overestimates the responses to fictive female stimuli. For LC11, the model accurately identifies LC11’s object selectivity (‘moving spot’) and suppression to loom and linear size. Similar to LC6, the 1-to-1 network overestimates LC11’s response magnitudes to the fictive female stimuli. We again found that for LC12, the 1-to-1 network has overly large responses to the fictive female stimuli but does predict magnitudes for moving spot, loom, and linear size. The model LC12 responses to loom and linear size appear to be inverted (i.e., flipping model LC12 responses to loom and linear size would better match the real LC12 responses)—this is likely a consequence of the fact that the sign of an LC’s response is unidentifiable for the 1-to-1 network, as one could simply flip the sign of the model LC unit’s response and the readout weights of downstream units. Other possible reasons are mentioned in **b**. Source data

**Extended Data Fig. 8:**
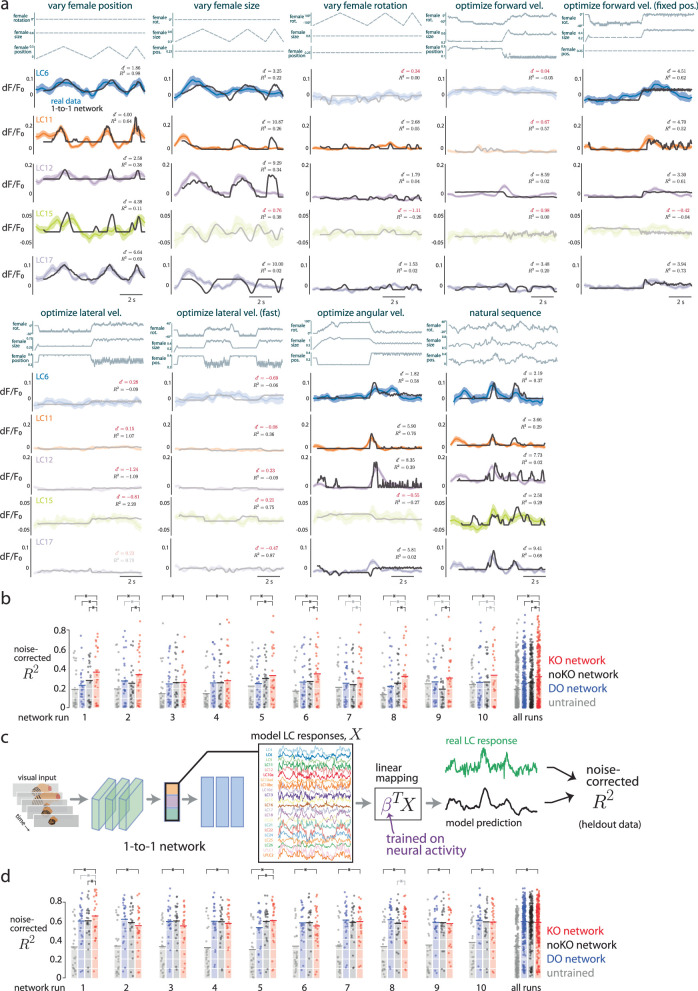
Real LC responses and predicted responses to stimulus sequences of a moving fictive female. **a**. We considered 9 different stimulus sequences in which a female varied her rotation, size, and position (three top traces for each stimulus sequence, see Methods for stimulus descriptions). We found that the 1-to-1 network’s predictions (black traces) largely predicted the responses of the real LC neurons (color traces), despite the facts that the 1-to-1 network was never given access to neural data and that we directly read out from a single model LC unit. The average of all reported noise-corrected *R*^2^s here is the same as that reported in Fig. 2e. We only considered stimulus sequences for which the real LC responses reliably varied across time for the stimulus sequence. To measure this, we computed the ${d}^{{\prime} }$ between splits of repeats (i.e., a signal-to-noise ratio across repeats) and considered any stimulus sequence with a ${d}^{{\prime} } < 1$ as unreliable, removing it from our analyses (translucent traces; see Methods). For some LC types, we detected a reliable response to only one or a few stimuli (e.g., LC15 only responded to ‘vary female position’ and ‘natural sequence’). We noticed that none of the LC neurons responded to stimulus sequences for which the fictive female’s parameters were chosen to optimize the 1-to-1 network’s output of lateral velocity (‘optimize lateral vel.’ and ‘optimize lateral vel. (fast)’, see Methods). This may be due to the fast changes in female position which were not present in other stimulus sequences. For each stimulus and LC type, we computed a noise-corrected *R*^2^ between the real and model predicted responses. This noise-corrected *R*^2^ overlooks any differences in mean, standard deviation, and sign of the response, which are unidentifiable by the KO network. For visual clarity, we centered, scaled, and flipped the sign of the 1-to-1 network predictions (black traces) to match the mean, standard deviation, and sign of the LC responses (color traces) for each stimulus. We accounted for the smoothness of calcium traces by applying a causal smoothing filter to the model LC responses as well as fitting the mean offset of the relu thresholding (see Methods). Interestingly, all LC types responded reliably to varying female position (‘vary female position’, color traces) despite the facts that the optic glomeruli have weak retinotopy^12,61^ and that the calcium trace is a sum of the activity of almost all neurons for the same LC type (presumably averaging away any spatial information). This suggests that either our targeted region for calcium imaging (Fig. 2a) was biased to read out from a subset of LC neurons with nearby receptive fields or that these LC neurons have some selectivity in female position (perhaps as direction selectivity). The latter may be more likely, as the male needs to better estimate female position than can be done simply by comparing coarse differences between the two optic lobes. Consistent with our findings, a previous study has identified another LC type—LC10a—to respond to an object’s position^11^. That our 1-to-1 network also predicted positional selectivity in the LC types (black traces) supports the notion that some optic glomeruli may track female position despite weak retinotopy. More work is needed to understand how object position is encoded within a single optic glomerulus and how that information is read out^61^. **b**. Results in **a **were for a KO network with one random initialization. To see if this effect holds for different initializations, we trained 10 runs of the KO network, each with a different random initialization and random ordering of training samples. We compared the runs of the KO network to those of the dropout (DO) network, for which a randomly-chosen model LC unit was dropped out during training, as well as noKO networks for which no knockout occurred during training. These are the same networks used to predict moment-to-moment behavior (Extended Data Fig. 3d). Each bar denotes the mean *R*^2^, and each dot denotes one combination of LC type and stimulus (i.e., non-shaded traces in **a**). Black asterisks denote a significant increase in mean *R*^2^ (*p* < 0.05, paired, one-sided permutation test, *n* = 27), gray asterisks denote a trend (*p* < 0.15). We observed that random initialization played more of a role for neural prediction than for behavioral prediction (Extended Data Fig. 3d). This is not so surprising, as the networks were never trained to predict neural responses. Still, the KO networks tended to outperform the other types of networks (red bars larger than other bars); combining across all runs, the KO network performed significantly better (‘all runs’, *p* < 0.002 for comparisons between KO and other networks, paired, one-sided permutation test, *n* = 270). In addition, the untrained networks performed poorly (gray bars), indicating that training networks on behavior did improve neural predictions. **c**. For the results in **a **and **b**, we considered a one-to-one mapping in which we directly compared a model LC unit’s response with real LC responses; our 1-to-1 network never had access to neural data for training. Here, we wondered if we relaxed this assumption (i.e., train a linear mapping from all model LC units to real LC responses), to what extent would the model’s prediction of real LC responses improve. The basic setup was the following. We feed a stimulus sequence into the 1-to-1 network (fully trained with knockout training) and collect responses from all model LC units, denoted as $X\in {{\mathcal{R}}}^{K\times T}$ for *K* model LC units (here, *K* = 23) and the *T* timepoints of the stimulus sequence. We then define a linear mapping $\beta \in {{\mathcal{R}}}^{K}$ to map the *K* model LC responses to the real LC response. We use real LC responses to train *β*. Specifically, for each of the 4 cross-validation folds, we train *β* on 75% of the real LC responses (randomly selected) using ridge regression. Training the linear mapping on responses to other stimuli led to worse performance, as expected, because the stimuli were largely different from each other—training on responses to a fictive female changing in position was not predictive of responses to a fictive female changing in size. We then predict the responses for the remaining held-out timepoints. We concatenate the predictions across the 4 folds and then compute the noise-corrected *R*^2^ in the same way as in Fig. 2d,e. Thus, the reported cross-validated noise-corrected *R*^2^s indicate the extent to which the 1-to-1 network, given neural data on which to train, can predict held-out real LC responses. Another view is that in this setting, the 1-to-1 network is a task-driven model trained on behavioral data with an internal representation (the model LC bottleneck) that reflects the activity of real LC neurons up to a linear transformation^1^. **d**. Prediction performance using the linear mapping for different networks and network runs (see Methods). For each network, we trained a new linear mapping between the model LC responses and the real LC responses. Overall, prediction performance greatly increased: The 1-to-1 network (or KO network) with the linear mapping had a noise-corrected *R*^2^ at ~ 65% (network run 1, averaged over all recorded LCs and fictive female stimulus sequences), an additive increase of ~ 30% over that of the 1-to-1 network with the one-to-one mapping comparison ( ~ 35%, Fig. 2e). We also found that, for the linear mapping, the performance of the 1-to-1 network was similar to those of the other networks trained with dropout (DO) or no knockout (noKO) (leftmost plot, red bar close to black and blue bars). This similarity in performance was not unexpected and indicates that all 3 networks (KO, DO, and noKO) have similar internal representations (up to a linear transformation) at the layer of their LC bottlenecks. However, the 1-to-1 network’s representation is better aligned to the LC types along its coordinate axes—where each model LC unit corresponds to one axis—than those of the other networks (Fig. 2e). Networks trained with behavioral data (KO, DO, and noKO) outperformed an untrained network (gray bar), indicating that training on behavior was helpful in identifying LC response properties. That the untrained network was somewhat predictive of LC responses (bar for ‘untrained’ above 0) stems from an inductive bias in which the network’s convolutional filters, even with randomized weights, can detect large changes of the visual stimulus (e.g., a fictive female moving back and forth). That a linear combination of random features is often predictive in a regression setting is a well-studied phenomenon in machine learning^62^ and has been observed in predicting visual cortical responses^63^. This trend in similarity of performance held across all 10 network runs (same runs as in **b**) for the different training procedures: The KO network consistently better predicted real LC responses than the untrained network but less so when compared to the DO and noKO networks (red bars at similar heights to black and blue bars across network runs). This trend held when combining across all runs (‘all runs’). A black asterisk indicates a KO network with a mean prediction performance significantly above that of another network (*p* < 0.05, paired, one-sided permutation test); a gray asterisk indicates a trend (0.05 < *p* < 0.15). Each bar denotes the mean *R*^2^, and each dot denotes one LC type and stimulus combination (i.e., the non-shaded traces in **a**); *n* = 27 for statistical tests for each run and *n* = 270 for all runs. Network run 1 was the chosen 1-to-1 network for Figs. 1–4. The results here indicate that by simply training a network on courtship behavioral data (i.e., a task-driven approach), we have identified a highly-predictive image-computable model of LC neurons. To our knowledge, ours is the first image-computable model of the LC population proposed. An important point is that this encoding model (using a linear mapping) does not identify a one-to-one mapping between model LC units and LC types, as the model is unable to relate the encoded LC neurons to behavior—this is precisely the reason we built the 1-to-1 network. Training the 1-to-1 network both on behavior and neural responses is a worthwhile goal, but care is needed to ensure the neural responses are recorded during natural behavior to achieve as best a match as possible. Source data

We computed this noise-corrected *R*^2^ between the 1-to-1 network and real responses for each LC type and stimulus sequence (Fig. 2e) for which the LC was responsive (that is, *d*′ > 1, see ‘Two-photon calcium imaging’). Importantly, the 1-to-1 network never had access to any neural data in its training; instead, for a given LC type, we directly took the response of the corresponding model LC unit as the 1-to-1 network’s predicted response. This is a stronger criterion than typical evaluations of DNN models and neural activity, where a linear mapping from DNN features (~10,000 feature variables) to neural responses is fit^1^. To account for the smoothness of real responses due to the imaging of calcium dynamics, we causally smoothed the predicted responses with a linear filter. We fit the weights of the linear filter (filtering the 10 past frames) along with the relu’s offset parameter (accounting for trivial mismatches due to differences in thresholding) to the real responses. This fitting only used responses of one model LC unit, keeping in place the one-to-one mapping; we also relaxed this constraint by fitting a linear mapping using all model LC units (Extended Data Fig. 8). We performed the same smoothing procedure not only for the 1-to-1 network but also for an untrained network, a network trained with dropout training, and a network trained without knockout (see ‘Knockout training’ above). This procedure was only performed for predicted responses in Fig. 2d,e and Extended Data Fig. 8. For analysing response magnitudes (Fig. 2f and Extended Data Fig. 7), the responses came directly from model LC units (that is, no smoothing or fitting of the relu’s offset was performed).

### Analysing model LC responses to visual input

To better understand how each model LC unit responds to the visual input, we passed natural stimulus sequences (taken from courtship sessions with control males) into the 1-to-1 network and computed the cross-validated *R*^2^ between model LC responses and each visual parameter (Fig. 3b). Because female position and rotation are circular variables, we converted each variable *x* to a 2D vector [cos(*x*),sin(*x*)] and took the maximum *R*^2^ across both variables for each model LC unit. We further investigated model LC tuning by systematically varying female size, position, and rotation to generate a large bank of stimulus sequences. We input these stimulus sequences into the 1-to-1 network and formed heat maps out of the model LC responses (Fig. 3c,d). For each input stimulus sequence, each of its 10 images was a repeat of the same image of a fictive female with a given size, lateral position, and rotation angle (that is, the fictive female remained frozen over time for each 10-frame input sequence). Across stimulus sequences, we varied female size (50 values linearly interpolated between 0.3 to 1.1), lateral position (50 values linearly interpolated between −1 to 1), and rotation angle (50 values linearly interpolated between −180 and 180 visual degrees), resulting in 50 × 50 × 50 = 125,000 different stimulus sequences that enumerated all possible combinations. To understand the extent to which each visual parameter contributed to a model LC unit’s response, we decomposed the total response variance into different components^37^ (Fig. 3e). The first three components represent the variance of the marginal response to each of the 3 visual parameters (which we had independently varied). We computed these marginalized variances by: (1) taking the mean response for each value of a given visual parameter by averaging the other two parameters over all stimulus sequences; and (2) taking the variance of this mean response over values of the marginalized parameter (50 values in total). Any remaining variance (subtracting the three marginalized variances from the total response variance) represents response variance arising from interactions among the three visual parameters (for example, the model LC response depends on female position but only if the female is large and faces away from the male, see Fig. 3d, ‘LC10a’). Because the 1-to-1 network was deterministic, no response variance was attributed to noise across repeats (unlike trial-to-trial variability observed in the responses of real neurons).

Analysing the model LC responses to a large bank of static stimuli is helpful to understand LC tuning (Fig. 3c–e). However, we may miss important relationships between the features of the visual input and model LC responses without considering dynamics (for example, the speed at which female size changes). To account for these other temporal features, we devised three dynamic stimulus sequences that varied in time for roughly 10 s each (Fig. 3f and Supplementary Video 2); these stimuli were similar to a subset of stimuli we presented to real male flies (see ‘Two-photon calcium imaging’). For each stimulus sequence, we varied one visual parameter while the other two remained fixed at nominal values chosen based on natural sequence statistics.

The first 2.5 s of each stimulus were the following:

1. vary female size: linearly increase from 0.5 to 0.9 with fixed position = 0 and rotation = 0°
2. vary female position: linearly increase from −0.25 to 0.25 with fixed size = 0.8 and rotation = 0°
3. vary female rotation: linearly increase from −45° to 45° with fixed size = 0.8 and position = 0

The next 2.5 s were the same as the first 2.5 s except reversed in time (for example, if the female increased in size the first 2.5 s, then the female decreased in size at the same speed for the next 2.5 s). Thus, the first 5 s was one period in which the female increased and decreased one parameter. The stimulus sequence contained 4 repeats of this period with different lengths (that is, different speeds): 5, 3.33, 1.66, and 0.66 s (corresponding to 150, 100, 50, and 10 time frames, respectively). We passed these stimulus sequences as input into the 1-to-1 network (that is, for each time frame, the 10 most recent images were passed into the model) and collected the model LC responses over time. We directly computed the squared correlation *R*^2^ between each model LC unit’s responses and the visual parameters (and features derived from the visual parameters, such as speed) for all three stimulus sequences (Fig. 3g). Velocity and speed were computed by taking the difference of the visual parameter between two consecutive time frames.

### Analysing how model LCs contribute to behaviour

Because the 1-to-1 network identifies a one-to-one mapping, the model predicts not only the response of an LC neuron but also how that LC neuron causally relates to behaviour. We wondered to what extent each model LC unit causally contributed to each behavioural output variable. We designed an ablation approach (termed the cumulative inactivation procedure (CLIP)) to identify which model LCs contributed the most to each behavioural output. The first step in CLIP is to inactivate each model LC unit individually by setting a model LC’s activity value for all time frames to a constant value (chosen to be the mean activity value across all frames). We found that setting the activity to 0 (as we do during knockout training) obscures nuanced but important relationships because a value of 0 may be far from the working regime of activity for a given stimulus, resulting in large deviations in predicted output. Instead, we focus on how variation in a model LC unit’s response contributes to variations in predicted behaviour. We test to what extent the 1-to-1 network with the inactivated model LC unit predicts the behavioural output of held-out test data from control flies (from the test set). We choose the model LC unit that, once inactivated, leads to the least drop in prediction performance (that is, the model LC unit that contributes the least to the behavioural output). We then iteratively repeat this step, keeping all previously inactivated model LC units still inactivated. In this way, we greedily ablate model LC units until only one model LC unit remains. After performing CLIP, we obtain an ordering of model LC units from weakest to strongest contributor of a particular behavioural output (Fig. 4b,c). We measure the contribution to behaviour as the normalized change in performance. For movement variables, normalized change in performance is the difference in *R*^2^ between no silencing (‘none’) and silencing *K* model LC units, normalized by the *R*^2^ of no silencing. For song variables, normalized change in performance is the same as for the movement variables except we use 1 − cross-entropy. We then use this ordering (and prediction performance) to infer which model LC units contribute to which behavioural outputs. We performed CLIP to predict held-out behaviour from control flies (Fig. 4c). Because different behavioural outputs had different prediction performances (Extended Data Fig. 3), we normalized each model LC unit’s change in performance by the maximum change in performance (that is, prediction performance for no inactivation minus that of inactivating all model LC units); for model LC units for which inactivation led to an increase in performance due to overfitting (Extended Data Fig. 12), we clipped their change in performance to be 1. We also performed CLIP to predict the model output to simple, dynamic stimulus sequences (Fig. 4d–f). Because we did not have real behavioural data for these dynamic stimulus sequences, we used the model output when no silencing occurred as ground truth behaviour.

### Connectome analysis

To obtain the pre- and postsynaptic partners of LC and LPLC neuron types, we leveraged the recently released FlyWire connectome of an adult female *Drosophila*^15,19^, for which optic lobe intrinsic neurons were recently typed^18^. We downloaded the synaptic connection matrix at https://codex.flywire.ai/ of the public release version 630. We isolated the following 57 LC and LPLC types: LC4, LC6, LC9, LC10a-f, LC11, LC12, LC13, LC14a1, LC14a2, LC14b, LC15, LC16, LC17, LC18, LC19, LC20a-b, LC21, LC22, LC24, LC25, LC26, LC27, LC28a, LC29, LC31a-c, LC33a, LC34, LC35, LC36, LC37a, LC39, LC40, LC41, LC43, LC44, LC45, LC46, LCe01-LCe09, LPLC1, LPLC2, and LPLC4. We report individual cell types LC10a, LC10b, LC10c, and LC10d which have been identified in FlyWire, but we do not yet know how the driver lines LC10ad and LC10bc map onto these individual types. We summed the number of synaptic connections across all neurons of the same type that were either inputs or outputs of one of the LC and LPLC neuron types. We denoted a connection (Fig. 5b, tick lines) if at least 5 synaptic connections existed between an LC or LPLC neuron type and another neuron type. We identified 538 presynaptic cell types and 956 postsynaptic cell types. We categorized partner cell types into classes based on the naming conventions in FlyWire’s connectome dataset^15^ and sorted cell types within each class based on the number of connections to the LC types. To see if LC types with similar inputs project to similar outputs—in other words, identify groupings of LC types, we performed agglomerative clustering separately on the pre- and postsynaptic connections. Specifically, we summed up connections across partner cell types within a class and used these summed connections as features for clustering (complete linkage with cosine similarity as affinity). LC types within a cluster are listed in numerical order. The following classes were used: LC, lobula columnar; LPLC, lobula plate-lobula columnar; AOTU, anterior optic tubercle; AVLP, anterior ventrolateral protocerebrum; CB, cross brain; CL, clamp; cL, centrifugal lobula; cM, centrifugal medulla; DN, descending neuron; Dm, distal medulla; Li, lobula intrinsic; LLPC, lobula-lobula plate columnar; LM, lobula medulla; LT, lobula tangential; mAL, medial antenna lobes; ML, medial lobe; MT, medulla tangential; OA, octopaminergic; PLP, posterior lateral protocerebrum; Pm, proximal medulla; PS, posterior slope; PVLP, posterior ventrolateral protocerebrum; SMP, superior medial protocerebrum; T2-T5, optic intrinsic; Tlp, translobula plate; Tm, transmedullary; TmY, transmedullary; Y, optic intrinsic; IB, inferior bridge; LAL, lateral accessory lobe; SAD, saddle; SLP, superior lateral protocerebrum; WED, wedge.

### Statistical analysis

Unless otherwise stated, all statistical hypothesis testing was conducted with permutation tests, which do not assume any parametric form of the underlying probability distributions of the sample. All tests were two-sided and non-paired, unless otherwise noted. Each test was performed with 1,000 runs, where *P* < 0.001 indicates the highest significance achievable given the number of runs performed. When comparing changes in behaviour due to genetic silencing versus control flies (Fig. 1e), we accounted for multiple hypothesis testing by correcting the false discovery rate with the Benjamini–Hochberg procedure with *α* = 0.05. Paired permutation tests were performed when comparing prediction performance between models (Fig. 2e) for which paired samples were randomly permuted with one another. Error bars of the response traces in Fig. 2b–d were 90% bootstrapped confidence intervals of the means, computed by randomly sampling repeats with replacement. No statistical methods were used to predetermine sample sizes, but our sample sizes are similar to those of previous studies^11,12,29,30^. Experimenters were not blinded to the conditions of the experiments during data collection and analysis.

### Reporting summary

Further information on research design is available in the Nature Portfolio Reporting Summary linked to this article.

## Online content

Any methods, additional references, Nature Portfolio reporting summaries, source data, extended data, supplementary information, acknowledgements, peer review information; details of author contributions and competing interests; and statements of data and code availability are available at 10.1038/s41586-024-07451-8.

### Supplementary information

Supplementary Tables

Reporting Summary

Supplementary Video 1 Video of 9 stimulus sequences of a fictive female used for evaluating the 1-to-1 network’s predicted LC responses versus real LC responses (Fig. 2 and Extended Data Fig. 8). Each stimulus sequence lasted for 10 s (300 frames). The 9 different stimulus sequences were the following: ‘vary female size’, ‘vary female position’, ‘vary female rotation’, ‘optimize for angular velocity’, ‘optimize for forward velocity’, ‘optimize for forward velocity with fixed position’, ‘optimize for lateral velocity’, ‘optimize for lateral velocity with multiple transitions’, and ‘natural stimulus sequence’. See Methods, section ‘Two-photon calcium imaging’ for specific details.

Supplementary Video 2 Video of the three simple stimulus sequences used to probe model LC tuning (Fig. 3) and model LC contributions to behavior (Fig. 4). Each stimulus sequence lasted 10 s (300 frames) and varied one of the fictive female’s parameters while keeping the remaining two fixed. The three stimuli are ‘vary female size’, ‘vary female position’, and ‘vary female rotation’. See Methods, ‘Analysing model LC responses to visual input’ for specific details.

### Source data

Source Data Fig. 1

Source Data Fig. 2

Source Data Fig. 3

Source Data Fig. 4

Source Data Fig. 5

Source Data Extended Data Fig. 1

Source Data Extended Data Fig. 2

Source Data Extended Data Fig. 3

Source Data Extended Data Fig. 4

Source Data Extended Data Fig. 5

Source Data Extended Data Fig. 6

Source Data Extended Data Fig. 7

Source Data Extended Data Fig. 8

**Extended Data Fig. 9:**
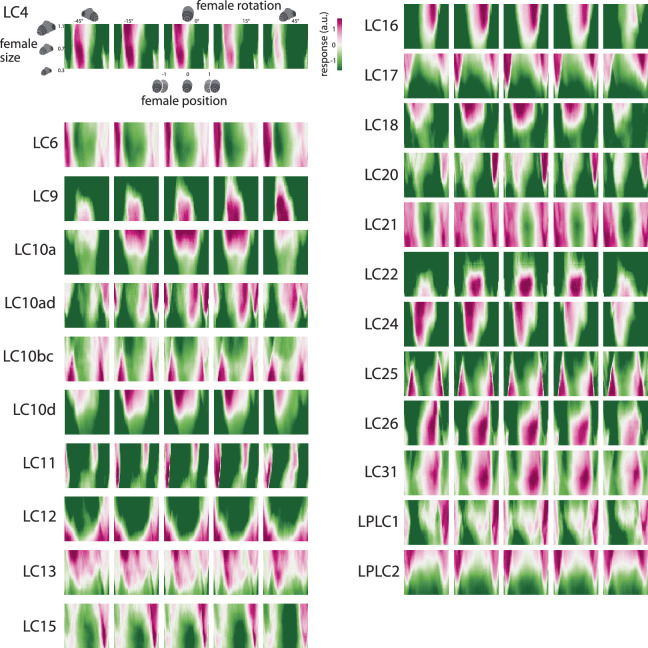
Model LC tuning heat maps. Each “pixel” in the heatmap corresponds to the response of the model LC unit to one input stimulus sequence in which a static fictive female fly has a given size, position, and rotation (i.e., all 10 images of the input sequence were the same, see Methods). We then systematically varied female size, position, and rotation across stimulus sequences (125,000 sequences in total). Same format as in Fig. 3c,d but for all model LC units. Source data

Source Data Extended Data Fig. 9

**Extended Data Fig. 10:**
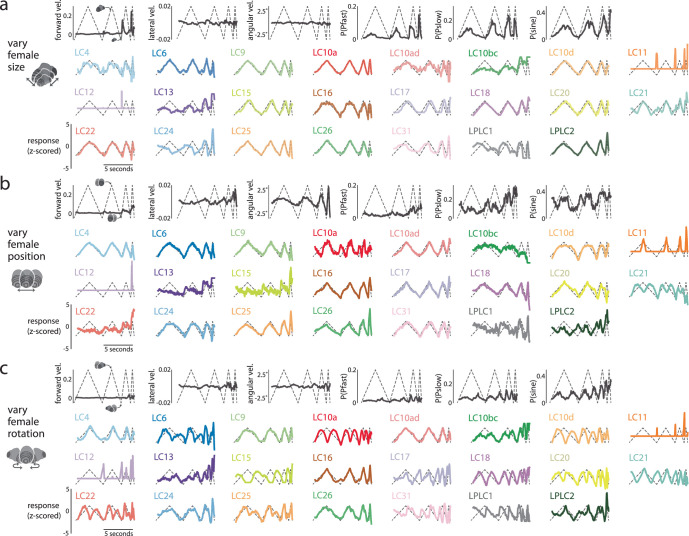
All model LC responses to simple, dynamic stimulus sequences in which only one visual parameter of the fictive female varied. Same dynamic stimulus sequences and format as in Fig. 3f; these responses were used to compute the *R*^2^s in Fig. 3g. We also show the 1-to-1 network’s behavioral output for each dynamic stimulus (top rows, black traces). Stimulus sequences include the following (see Methods for exact parameter values): **a**. Varying female size while the female stays in the middle facing away from the male. **b**. Varying female position while the female has a fixed, large size and faces away from the male. **c**. Varying female rotation while the female has a fixed, large size and stays in the middle. Each trace’s sign was flipped to have a positive correlation with the varying visual parameter of the corresponding stimulus sequence. Source data

Source Data Extended Data Fig. 10

**Extended Data Fig. 11:**
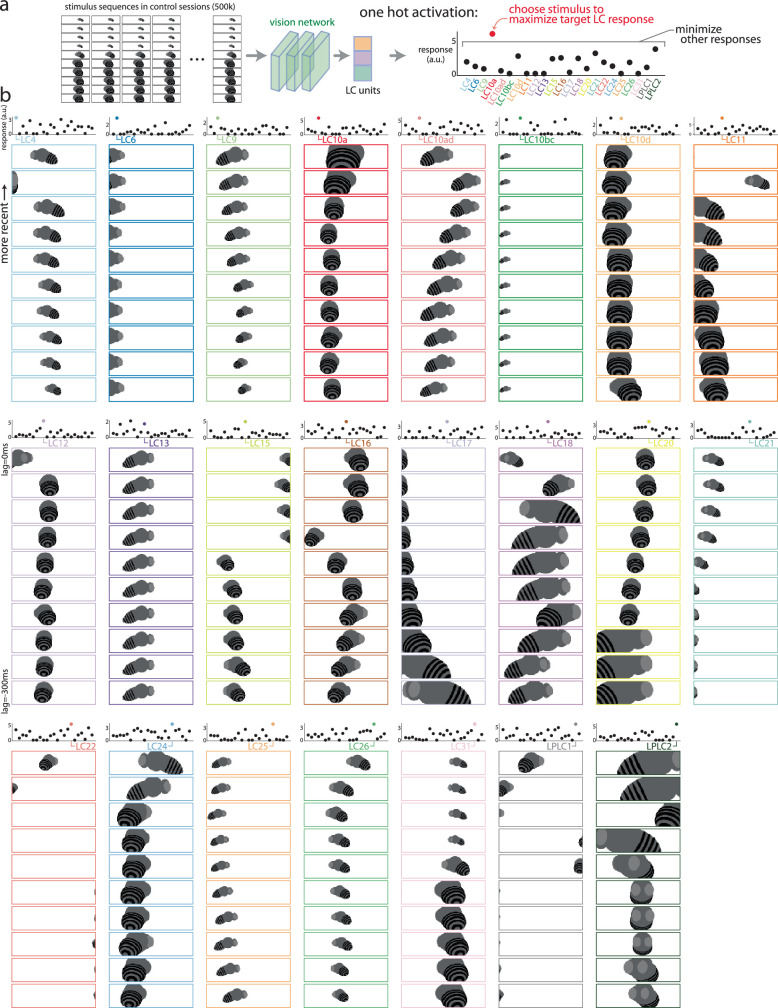
Maximizing visual inputs for each model LC unit. To better understand the differences in stimulus preference across the model LC units, we optimized the visual input history that maximized each model LC unit’s response while minimizing responses of all other model LC units (i.e., a ‘one-hot’ maximizing stimulus). **a**. We considered a large number of candidate stimulus sequences taken from the training dataset of control sessions (500,000 stimulus sequences in total). We fed each stimulus sequence as input into the 1-to-1 network, extracting the responses of the model LC units. We chose the stimulus sequence that maximized a chosen model LC unit’s response while minimizing the responses for all other model LC units. We used the following objective function *f*_*i*_(**x**) for the *i*th chosen model LC unit, adopted from^64^: ${f}_{i}({\bf{x}})=\frac{\exp ({r}_{i}({\bf{x}}))}{{\sum }_{j\ne i}\exp ({r}_{j}({\bf{x}}))}$ where **x** is the visual input sequence of 10 frames and *r*_*i*_ is the response of the *i*th model LC unit. The objective function *f*_*i*_(**x**) is maximized for large responses of the *i*th model LC unit and responses as small as possible for all other units. Thus, we optimize stimulus sequences as “one-hot maximizations”. **b**. Maximizing stimulus sequences for each model LC unit with the most recent frame as the top image. One hot maximization worked for a handful of model LC units (LC9, LC10a, LC11, LC12, LC15; top panel shows responses of all model LC units to that stimulus sequence); surprisingly, one-hot maximization failed to drive a single model LC unit for many of the other LC types (at least one black dot has similar value to color dot), indicating that these model LC units share stimulus preferences with other model LC units. Some stimulus sequences have smooth changes to the fictive female’s parameters, such as LC10a and the increase in female size. However, other maximizing stimulus sequences show large jumps of the fictive female (e.g., LC4, LC11, LC12, LC22, etc.); even though these stimulus sequences were chosen from natural courtship, they likely represent outliers that strongly drive responses. This is especially true of model LC11 that prefers a small female moving at a fast speed, consistent with LC11 being a small object detector^27,28^. These maximizing stimulus sequences represent predictions of the 1-to-1 network that can be tested in future experiments to see if they truly elicit large responses from LC neurons, much like recent work has identified images to drive visual cortical neurons of macaque monkey^64–67^. Other objective functions, such as maximizing the response variation across time with a longer stimulus sequence, and other constraints, such as restricting how much a fictive female may change between consecutive frames or requiring the fictive female to not remain static, are easily possible with the 1-to-1 network. Our main finding here is that many of the one-hot maximizing stimuli failed to only activate the targeted LC type; this is further evidence that visual features are distributed across the LC population. Source data

Source Data Extended Data Fig. 11

**Extended Data Fig. 12:**
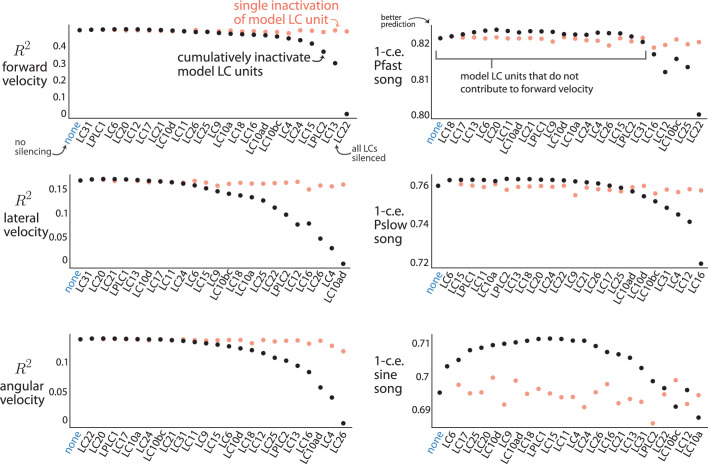
Inactivating model LC units for each of the 6 behavioral output variables during natural courtship. We inactivated each model LC unit separately and re-computed the predicted performance *R*^2^ or 1-c. e. (cross-entropy) on held-out behavioral data from control flies (red dots). We inactivated each model LC unit by setting its activity equal to its time-averaged response; we found this approach better able to tease apart LC contributions versus setting activity to 0 (i.e., what is done during knockout training), as the latter often leads to changes in mean behavior but does not alter the moment-by-moment sensorimotor transformation (see Methods). Inactivating any single model LC unit did not lead to a large drop in performance, consistent with our experimental findings (Extended Data Fig. 1). This indicates that only by inactivating multiple model LC units at the same time will we see a deficit in prediction; in other words, the behavior relies on reading out from combinations of LC types. To identify these combinations, we inactivated model LC units in a cumulative, greedy manner (black dots) and observed to what extent the responses for the remaining model LC units predict held-out behavioral data from control flies. Same format as in Fig. 4b. For each plot, model LC units on the left contribute the least to the given behavioral output; model LC units on the right contribute the most. We found that when inactivating some model LC units, performance actually *increased* (e.g., LC12 for sine song, bottom right). This is because LC10bc and LC12 used excitation and inhibition to cancel out some of each other’s responses—ablating one decreases performance while ablating both increases performance as both excitatory and inhibitory effects are removed via ablation. The LC neurons themselves need not be either excitatory or inhibitory; readouts by downstream neurons may rely on positively or negatively weighting the LC responses. Performance also increased by removing a number of model LC units for sine song (bottom right, LC6); this is possibly due to overfitting by the 1-to-1 network. By removing “noisy” model LC units that are overfit to the training data, the rest of the model LC units better generalize. Interestingly, the strongest contributor of the model LC units, if inactivated alone, did not lead to a large decrease in performance. For example, LC22 was the strongest contributor for forward velocity but, when inactivated alone, resulted in little decrease to *R*^2^ (red dot above black dot). This is consistent with our finding that silencing LC22 led to little change in mean forward velocity (Extended Data Fig. 1). We note that inactivating any single model LC unit will likely lead to changes in forward velocity in specific contexts which the 1-to-1 network identifies (Extended Data Fig. 3); here, for simplicity we compute *R*^2^ across aggregated contexts for the entire courtship session of control males and potentially miss changes in specific contexts. That inactivating any single model LC unit leads to little drop in performance suggests that the model LC units work together as a population code to sculpt behavior: There is no sole contributor to any particular behavior, especially when combining across different behavioral contexts (e.g., chasing the female from far away, singing to a nearby female, etc.) as is done here. The red squares of the heatmaps in Fig. 4c (which condense the information plotted here) correspond to the differences between the performance value (*R*^2^ or 1-c. e.) for each model LC unit and no inactivation (‘none’), divided by the maximum difference (in most cases, the difference between the value for the rightmost model LC and the value for ‘none’). To avoid the effects of overfitting, any positive differences (i.e., an increase in prediction performance) were clipped to 1. Source data

Source Data Extended Data Fig. 12

## Data Availability

Data are available at https://dandiarchive.org/dandiset/000951/. Source data are provided with this paper.

## Code availability

The code for extracting fly body positions (SLEAP) is available at https://sleap.ai/. Song segmentation was performed with code found at https://github.com/murthylab/MurthyLab_FlySongSegmenter. Model weights, example stimuli and code are available at https://github.com/murthylab/one2one-mapping. The FlyWire connectome is available at https://codex.flywire.ai/.
