# Supplementary material for: Mapping model units to visual neurons reveals population code for social behaviour
Source: Nature. 2024 May 22;629(8014):1100–8. doi: 10.1038/s41586-024-07451-8 (PMC11136655; doi:10.1038/s41586-024-07451-8)
Supplement: Supplementary file 1 — Supplementary Tables [file 41586_2024_7451_MOESM1_ESM.pdf]

nature portfolio

https://doi.org/10.1038/s41586-024-07451-8

Supplementary information

Mapping model units to visual neurons reveals population code for social behaviour

In the format provided by the authors and unedited

cell type	driver line	AD in *attP40* (or VK00027)	DBD in *attP2*
control	Empty_Split-Gal4 (BL79603)	-	-
LC4	SS00315	R47H03	R72E01
LC6	OL0077B	R92B02	R41C07
LC9	SS02651	VT032961	VT040569
LC10a	OL0019B	R35D04	R22D06
LC10ad	OL0020B	R35D04	R80E07
LC10bc	OL0023B	R72C08	R50D07
LC10d	SS03822	VT043920	R91B01
LC11	OL0015B	R22H02	R20G06
LC12	OL0008B	R35D04	R55F01
LC13	OL0027B	R14A11	R50C10
LC15	OL0042B	R26A03	R24A02
LC16	OL0046B	R26A03	R54A05
LC17	OL0005B	R21D03	R65C12
LC18	OL0010B	R92B11	R82D11
LC20	SS00343	R17A04 (VK00027)	R35B06
LC21	OL0044B	R55C04	R85F11
LC22	OL0001B	R64G10	R35B06
LC24	SS02638	VT038216	VT026477
LC25	SS02650	VT009792	VT002021
LC26	SS02445	VT007747	R85H06
LC31	SS04926	VT038873	VT025493
LC31	SS05022	VT045617	VT025493
LPLC1	OL0029B	R64G09	R37H04
LPLC2	OL0048B	R19G02	R75G12

Supplemental Table 1. Genetic line information for each of the LC types. ALL LC lines (Wu et al. eLife) and the spGAL4 control line were generously provided by M. Reiser, A. Nern, and G. Rubin. The LC31 lines were generously provided by A. Nern and G. Rubin ahead of publication (Nern et al., *in prep.*). The empty stable split GAL4 line (spGAL4) was used as a control line (BL79603): w[1118]; Py[+t7.7] w[+mC]=p65.AD.UwattP40; Py[+t7.7] w[+mC]=GAL4.DBD.UwattP2 [**?**]. Males were paired with females of the following genotype: PIBL (pheromone-insensitive and blind) *w+/w+; GMR-hid/+; orco/ orco* (recombined into the CS background, see below)

In Extended Data Figure **??**, the CS control data was from a previous study [**?**], listed as Wildtype 2, Canton S laboratory strain. Blind males (BL) were of the following genotype and also recombined into the Canton S background: w+/w+; GMR-hid/GMR-hid, also from the same previous study [**?**].

More information can be found at the following websites:

- `https://splitgal4.janelia.org/precomputed/LC%20Paper.html`
- `https://flybase.org/reports/FBst0028996.html`
- `https://flybase.org/reports/FBtp0001264.html`

cell type	driver line	AD in *attP40*	DBD in *attP2*
LC6	OL0070B	R91B01	R22A07
LC11	OL0015B	R22H02	R20G06
LC12	OL0008B	R35D04	R55F01
LC15	OL0042B	R26A03	R24A02
LC17	OL0005B	R21D03	R65C12

Supplemental Table 2. We crossed each of the 5 LC split lines to GCaMP6f (+/+; UAS GCaMP6f/CyO; +, provided by the Clandinin Lab) and imaged from the male progeny.
